# Physiological serum uric acid concentrations correlate with arterial stiffness in a sex-dependent manner

**DOI:** 10.1186/s12916-025-04195-8

**Published:** 2025-07-01

**Authors:** Oliver Thews, Thomas Schmid, Alexander Kluttig, Andreas Wienke, Melanie Zinkhan, Wolfgang Ahrens, Till Bärnighausen, Hermann Brenner, Stefanie Castell, Berit Lange, Wolfgang Lieb, Karin Halina Greiser, Marcus Dörr, Lilian Krist, Stefan N. Willich, Volker Harth, Nadia Obi, Michael Leitzmann, Annette Peters, Börge Schmidt, Matthias B. Schulze, Henry Völzke, Matthias Nauck, Stephanie Zylla, Anke Hannemann, Tobias Pischon, Ilais Moreno Velásquez, Matthias Girndt, Claudia Grossmann, Michael Gekle

**Affiliations:** 1 Julius-Bernstein-Institut für Physiologie, Martin-Luther-Universität Halle-Wittenberg, Halle (Saale), Germany; 2 AG Digitale Forschungsmethoden in der Medizin, Martin-Luther-Universität Halle-Wittenberg, Medizinische Fakultät, Halle (Saale), Germany; 3 Studienzentrumder, NAKO Gesundheitsstudie , Halle (Saale), Germany; 4 Institute for Medical Epidemiology, Biometrics and Informatics, Martin Luther University Halle-Wittenberg, Halle (Saale), Germany; 5 Biobank der Universitätsmedizin Halle, Martin-Luther-Universität Halle-Wittenberg, Halle (Saale), Germany; 6 Department Epidemiological Methods and Etiological Research, Leibniz Institute for Prevention Research and Epidemiology—BIPS , Bremen, Germany; 7 Medical Faculty and University Hospital, Heidelberg Institute of Global Health (HIGH), Heidelberg University, Heidelberg, Germany; 8 Department of Global Health and Population, Harvard T.H. Chan School of Public Health, Boston, USA; 9 Harvard Centre for Population and Development Studies, Cambridge, MA USA; 10 Division of Clinical Epidemiology and Aging Research, German Cancer Research Center (DKFZ), Heidelberg, Germany; 11 Department for Epidemiology, Helmholtz Centre for Infection Research, Brunswick, Germany; 12 Institute of Epidemiology, Kiel University, Kiel, Germany; 13 Division of Cancer Epidemiology, German Cancer Research Centre, Heidelberg, Germany; 14 Department of Internal Medicine B, Angiology & Pneumology, University Medicine Greifswald, CardiologyGreifswald, Germany; 15 German Centre for Cardiovascular Research (DZHK), Partner Site Greifswald, Greifswald, Germany; 16 Institute of Social Medicine, Epidemiology and Health Economics, Charité-Universitätsmedizin Berlin, Berlin, Germany; 17 Institute for Occupational and Maritime Medicine (ZfAM), University Medical Center Hamburg-Eppendorf, Hamburg, Germany; 18 Institut für Epidemiologie Und Präventivmedizin, Universität Regensburg, Regensburg, Germany; 19 Institute of Epidemiology, Helmholtz Zentrum München—German Research Center for Environmental Health (GmbH) , Neuherberg, Germany; 20 Institute for Medical Informatics, Biometry and Epidemiology, University Hospital of Essen, University of Duisburg-Essen, Essen, Germany; 21 Department of Molecular Epidemiology, German Institute of Human Nutrition Potsdam-Rehbruecke, Nuthetal, Germany; 22 Institute of Nutritional Science, University of Potsdam, Nuthetal, Germany; 23 Institut für Community Medicine, Abteilung SHIP/Klinisch-Epidemiologische Forschung, University Medicine Greifswald, Greifswald, Germany; 24 Institut für Klinische Chemie Und Laboratoriumsmedizin, University Medicine Greifswald, Greifswald, Germany; 25 Max-Delbrueck-Center for Molecular Medicinein the, Molecular Epidemiology Research Group , Helmholtz Association (MDC), Berlin, Germany; 26 Max-Delbrueck-Center for Molecular Medicinein the , Helmholtz Association (MDC), Biobank Technology Platform, Berlin, Germany; 27 Charité - Universitätsmedizin Berlin, Freie Universität Berlin and Humboldt-Universität zu Berlin, Berlin, Germany; 28 Universitätsklinik für Innere Medizin II, Martin-Luther-Universität Halle-Wittenberg, Halle (Saale), Germany; 29 Institute for Medical Information Processing, Biometry and Epidemiology, Medical Faculty, Ludwig-Maximilians-Universität München, Munich, Germany; 30 DZHK (German Centre for Cardiovascular Research), Partner Site Munich Heart Alliance, Munich, Germany

**Keywords:** Urate, Vascular stiffness, Vascular damage, Pulse wave velocity, Hyperuricemia, Female health

## Abstract

**Background:**

In humans, uric acid is a product of purine metabolism that impacts the vascular system. In addition to effects on arterial vascular tone, associations between serum uric acid concentrations—even in the physiological range—and arterial hypertension and vascular-mediated end-organ damage due to an impact on vascular stiffness have been postulated.

**Methods:**

Therefore, we aim to investigate a possible cross-sectional association between serum uric acid concentrations in the physiological range and differences in arterial pulse wave velocity (PWV), an indicator of vascular remodeling, with a focus on possible differences between female and male individuals. We analyzed cross-sectional phenotypic and laboratory parameters, including PWV from 70,649 individuals in the population-based German National Cohort (NAKO) in a sex-specific manner. In parallel, we applied a machine learning approach to identify and quantify factors associated with PWV in a hypothesis-free manner.

**Results:**

Our analysis uncovered a positive association between serum uric and PWV which was detected even if only individuals with urate values in the physiological range were included (*n* = 64,095). This correlation was more pronounced in women than in men. In multivariable linear regression models, we observed an association of uric acid (mmol/l) with PWV (m/s) of *β* = 1.12 (95% confidence interval (CI): 0.78; 1.45) in males and *β* = 1.35 (1.05; 1.66) in females, independent of other factors known to affect vascular stiffness. In addition, the machine learning approach identified uric acid as a major factor associated with PWV. The positive association was not restricted to hyperuricemia but evident even in the physiological concentration range. Based on the data from studies on the impact of aging on PWV, it is estimated that an increase in serum uric acid concentration by 0.1 mmol/l corresponds to an increase of approx. 7 years of age in females and of 4 years in males.

**Conclusions:**

Already in the physiological concentration range, uric acid is positively associated with parameters of arterial stiffness. This association is more pronounced in females as compared to males. This finding provides a mechanistic explanation for the increased risk of vascular end-organ damage associated with higher serum uric acid concentrations and supports the observed greater benefit of therapeutic uric acid lowering in female. Future intervention studies have to address the mechanistic causality of the observed effect.

**Supplementary Information:**

The online version contains supplementary material available at 10.1186/s12916-025-04195-8.

## Background

Cardiovascular diseases are a still growing clinical and public health problem, with established risk factors but also additional influencing factors that are poorly understood, like uric acid. Unlike in many other mammals, in humans, uric acid is the final product of endogenous and dietary purine metabolism due to an evolutionary loss of uricase activity. As a weak acid, the majority of uric acid circulates in the blood as urate. When the solubility limit of around 360–420 µmol/l [1–3] is exceeded, urate crystals may form and precipitate. It is estimated that ~ 700 mg urate per day have to be excreted by adults, mainly by the kidneys, to maintain the steady-state concentrations [1]. Of note, 500 to 600 mg per day are produced endogenously predominantly in the liver and to a minor part in the small intestine. A crucial metabolic step during urate formation is mediated by xanthine oxidase at the end of the purine pathway [4]. Diet can enhance the urate load by either direct uptake of nucleotides or enhanced ingestion of fructose that enhances adenosine monophosphate formation [1].

Physiological uric acid concentrations have been assessed in individuals without evidence of gout to range between 210 and 420 µmol/l in adult males and postmenopausal women and between 160 and 360 µmol/l in premenopausal women [5]. However, the meaning of these cut-offs is under continuous debates, as there is evidence that uric acid might play a pathophysiological role in cardiovascular and renal disorders even in concentrations lying within the physiological range. It has been suggested that an upper threshold value of 360 µmol/l should be considered for all individuals [5].

In human kidney, urate is freely filtered in the glomeruli but reabsorbed to more than 90% in the proximal tubule [6], mainly by the apical transporter URAT1 [1, 6–8]. In parallel, urate is also secreted into the proximal tubule, leading to a fractional excretion of ~ 10%. URAT1 is an important determinant of reabsorption and is the target of uricosuric drugs such as probenecid [7, 8]. Long-term treatment with diuretics at higher doses can reduce urate excretion, either by enhanced reabsorption in the proximal tubule or competition for secretory mechanisms in proximal tubule cells [9]. Hyperuricemia results in more than 90% of the cases from an inappropriate renal excretion [1, 2, 10], defined as the shift towards higher steady-state serum uric acid concentrations when handling the daily incoming urate [11].

Uric acid was initially considered a waste product [1], but several studies showed that it may exert beneficial biological effects. Uric acid has been reported to act either as antioxidant or as pro-oxidant and contributes substantially to the antioxidant capacity of human serum [12]. Furthermore, it can prevent protein nitrosylation, lipid peroxidation, or tetrahydrobiopterin inactivation [6]. On the other hand, uric acid can promote sodium retention and high blood pressure [13], an advantage during an evolutionary period in which sodium was scarce [14, 15]. Also, it has been reported to contribute to the development of insulin resistance and obesity [16–18]. Finally, even beneficial cognitive effects and strengthening of immune functions have been proposed [19–22]. These evolutionary advantageous actions of uric acid are contrasted by data suggesting pathophysiological effects, most prominently gout, but also cardiovascular, renal, and metabolic dysfunctions [2, 23]. Thus, uric acid cannot be regarded simply as harmful or beneficial. There seems to be a collision between its evolutionary function and its additional impact on human physiology due to socioeconomic wealth with the dramatically altered availability of, e.g., salt and meat in modern times. These marked transition adjustments led to increased physiological serum uric acid levels and could represent a non-beneficial adaptation [15].

Epidemiological studies have reported relations between serum uric acid levels and cardiovascular risk factors, as reviewed by Feig et al. [24–28]. Hyperuricemia is associated with an increased risk of hypertension, independent of other risk factors, an effect which was found to be more pronounced in women than in men [29, 30]. Treatment of hypertensive adolescents with uricosurica was reported to be associated with a reduction of blood pressure [31]. Pathologically elevated uric acid concentrations are also associated with the metabolic syndrome [32] and elevated urate levels have been reported to precede insulin resistance [33, 34] and obesity [35]. High levels of serum uric acid were suggested as an independent risk factor for developing type 2 diabetes [36, 37]. However, it is unclear whether this association is causal [38]. Pathophysiologically, hyperuricemia-induced endothelial dysfunction seems to play an important role [23, 24]. Regarding uric acid levels and cardiovascular mortality, there is a relationship that is more pronounced in women compared to men [39]. Also, the mortality due to ischemic heart disease is five times higher in women with serum uric acid > 420 µmol/l compared to women with values < 240 µmol/l, whereas there was no difference in the risk ratio for men [40]. Recently, a large cohort study reported the association of gout (crystal arthropathy due to hyperuricemia) with 12 cardiovascular diseases and a greater risk for women than for men [41]. The importance of uric acid, even in the “normal” concentration range, as a cardiovascular risk factor is highlighted by several other studies [42–48]. Additionally, the uric acid levels may be a modifiable risk factor and pharmacological lowering of uric acid concentrations may reduce the risk of cardiovascular events [49–52].

Many studies show that a person’s sex can influence their risk of disease and subsequent outcome when it comes to, e.g., cardiovascular conditions, making the need and demand obvious to include females and males in pathophysiological and clinical investigations [53]. Therefore, we put a special focus on the comparison of data from female and male individuals. The correlation of the arterial pulse wave velocity (as a measure of vascular stiffness) and the actual serum urate concentration was analyzed for females and males separately.

Although several studies about uric acid as a cardiovascular risk factor already exist, the results are not conclusive and often neglect the effect of uric acid levels in the normal range. Additionally, sex differences are rarely explored. The aim of the present study is to analyze the relationship of physiological serum uric acid concentrations with vascular stiffness as an indicator of vascular remodeling in human females and males from the general population, independent of blood pressure. Such a relationship could close the knowledge gap in understanding mechanisms linking the described impact of urate on vascular cells and the epidemiological evidence for urate as risk factor for vascular end-organ damage.

In parallel, we applied a machine learning (random forest, RF) approach in the sense of a plausibility and sensitivity analysis. The classic selection of variables for the linear regression model was nevertheless made based on preliminary considerations about possible confounders. Therefore, the RF analysis does not represent the main result of this study.

## Methods

### Study sample

The German National Cohort (NAKO) is a prospective, population-based cohort study in which 205,415 participants aged 19–74 in 18 study centers were recruited between 2014 and 2019. Random samples were drawn in the study regions via the residents’ registration offices. The inclusion criteria were age 19–69 at the time of sampling and a main residence in the study region. Only people who were unable to come to the study center and people who did not have sufficient knowledge of German language were excluded. In such cases, participation was only possible if a translator was available for study information, explanation, consent form, and explanation of the study modules. Recruitment was stratified by age and sex, whereby the older age groups (> 40 years) were oversampled.

NAKO aims to investigate the development and etiology of diseases, and to identify risk factors and enhance early detection and prevention of diseases with a focus on diabetes, cancer, cardiovascular, pulmonary, neurological, psychiatric, and infectious diseases. All participants gave their written informed consent. The responsible ethics committees approved the study [54, 55]. For the present analyses, data from the first 101,556 participants who had completed the baseline examination (data-freeze 100k) were available with the necessary quality control (Additional file 1: Fig. S01).

### Data collection and handling

General information regarding the NAKO study design and methods are described elsewhere [54, 55]. In brief, during baseline examination, participants were examined intensively, including a face-to-face interview, self-administered, computer-based questionnaires, broad biomedical examinations (e.g., measurement of blood pressure, grip strength, spirometry, anthropometric measures, vascular status), and provision of bio-samples (i.a., blood, urine). A 20% random sample underwent extended examinations such as additional imaging (e.g., ultrasonography for abdominal fat).

### PWV determination

Arterial pulse wave velocity (PWV) was recorded and calculated by Vascular Explorer (Enverdis GmbH, Jena, Germany) [56]. Subjects were placed in a supine position for at least 10 min before starting the assessment. Pulse wave analysis as well as ankle and brachial blood pressure was automatically calculated by the software based on the photoplethysmographic signal from the fingers and toes and the pressure changes in the cuffs. All subjects were asked to avoid speaking and encouraged to breathe calmly during the measurements. For our analysis, we used the parameter pwv_ba(2) = (La − Lb)/PTT (m/s). PTT = pulse transit time; La = jugular to ankle cuff distance; Lb = jugular to brachial cuff distance.

### Relevant measurement procedures

Serum urate levels were photometric measurements on different platforms, depending on the commissioned laboratory by the local study center (Dimension Vista 1500 (Siemens Healthineers, Erlangen, Germany), Advia 2400 (Siemens Healthineers, Erlangen, Germany), AU5822 (Beckman Coulter, Brea, USA), AU680 (Beckman Coulter, Brea, USA), DxC 800 (Beckman Coulter, Brea, USA), Cobas 8000 (Roche Diagnostics, Rotkreuz, Switzerland), Cobas c502 (Roche Diagnostics, Rotkreuz, Switzerland), Cobas c701 (Roche Diagnostics, Rotkreuz, Switzerland)). Plasma levels of HbA1c and serum levels of HDL cholesterol, LDL cholesterol, and creatinine were measured by certified standard techniques in the central laboratories of the hospitals participating in NAKO or in laboratories commissioned by the study centers.

Body mass index (BMI) was calculated as weight (kg) divided by height in meters squared (m^2^), whereby height and weight were measured with the Seca stadiometer 274 (Seca, Hamburg, Germany). The blood pressure was measured oscillometrically twice on the right arm with the OMRON HEM-705CP device (Omron, Kyoto, Japan) after a 5-min rest period. The second measurement was used for the analysis. Smoking history, alcohol intake, and physical activity were collected as part of the standardized touchscreen-based survey. The intake of medication was recorded in a face-to-face interview. All medications taken in the last 7 days were registered using special software. Participants who had taken medication of the ATC classes C02, C03, C07, C08, and C09 in the last 7 days were classified as anti-hypertensive drug users. This includes also all kinds of diuretics (ATC code C03) which may affect blood pressure but also urate excretion. Since lipid metabolism can also affect vascular stiffness, taking of lipid-lowering drugs (ATC code C10) was also documented. Finally, urate-lowering drugs (ATC code M04) (which includes uricostatics and uricosurics) were also included in the analyses.

### Statistical analysis

#### Linear models

In a multiple linear regression model, we also included several other variables known to be associated with vascular remodeling and urate concentration. The distribution of all variables in the study sample [57] was described by adequate parameters as recommended (continuous variables: mean, median, standard deviation, range; qualitative variables: frequency distribution). The association between the arterial pulse wave velocity (outcome variable/dependent variable) and a set of the independent variables (serum urate, sex, age, body mass index, smoking history, alcohol intake, HbA1c, HDL cholesterol, LDL cholesterol, serum creatinine, physical activity, systolic blood pressure, and treatment with anti-hypertensive drug (including diuretics), with lipid-lowering or urate-lowering drugs) was analyzed by a multiple linear regression model. From the complete number of individuals in the NAKO 100k data freeze (*n* = 101,556), the regression analysis was performed for all individuals with the complete dataset concerning the analyzed variables (*n* = 70,649). The reduction of the number of complete samples was mostly due to missing values of the PWV (Vascular Explorer™ measurements; ~ 11,000 missings), of the LDL cholesterol (~ 10,000 missings), HDL cholesterol, HbA1c, and alcohol intake (each ~ 6000 missings). In addition, the analysis was performed separately for female and male participants as well as for participants with normal serum urate levels (male: ]180, 420] µmol/l; female: ]140, 360] µmol/l).

The information on sex is numerically coded with 1 = male, 2 = female and the regression is calculated using this numerical information. Thus, if the regression coefficient for PWV is negative, it means that women (“2”) have a smaller PWV than men (“1”).

To analyze interaction of independent variables also models with interaction terms (e.g., sex and urate level) were calculated. The results of the regression analyses were expressed by the regression coefficients *β* with their confidence interval and the respective *p* value.

#### Non-linear models (machine learning)

In order to assess the relative importance of a wide range of independent variables with PWV, allowing for non-linear associations and possibly interactions, we applied the random forest algorithm [58] to build a non-linear high-dimensional regression model for PWV using the “randomForest” package in R, version 4.7–1.1, which provides a built-in feature importance assessment (https://cran.r-project.org/web/packages/randomForest/index.html).

This approach differs from the linear analysis (see previous section) because random forests are non-linear by definition and the regression achieved is accordingly non-linear. Note that the assumption of linear models is an additional outcome of our analysis—but not an a priori condition. It is not unusual to initially assume a non-linear relationship before narrowing the focus to a linear model.

In preparation for this task, several data preprocessing steps were undertaken. Firstly, observations with missing values in the target variable (PWV) were excluded (*n* = 11,269). Next, variables with more than 5000 missing entries (approximately 5% of samples) were removed (see Additional file 2) to eliminate features with excessive missing data that could introduce bias or noise. Also variables which are directly strongly correlated with parameter in the model were removed (e.g., therapy for a specific disease and existence of the disease itself). For this reason, all variables obtained using the Vascular Explorer™, except PWV (see Additional file 2), were removed to avoid implicit redundancy. Variables containing non-relevant information, such as study center site and metadata for measurement values (like the used platform), were also removed to eliminate potential noise or redundancy. A final step removed any remaining observations with missing values in the remaining variables. The resulting dataset consisted of 38 variables (Additional file 2: Tables S01 and S02) and 74,495 observations, providing a clean training dataset.

For reliable model evaluation of the random forest training, we adopted a tenfold cross-validation (CV) training. Each of the ten models was trained with 500 decision trees, aggregating individual outputs for stable predictions. At each split, 22 variables were randomly sampled as candidates, promoting tree diversity and capturing different data aspects. Terminal nodes grew without pruning, with a minimum size of 1 observation. Cases were sampled with replacement (bootstrapping) to grow each tree. The full training data was used to grow each tree. Equal priors were assigned to classes in the classification task. Feature importance results of this CV training scheme are reported by mean and standard deviation; for results of individual folds see Additional file 2: Fig. S02 and Table S02.

All calculations were performed with SPSS Ver. 28 (IBM, Armonk, NY, USA) and in R, version 4.7–1.1.

### Ethics approval

The study was approved by the responsible local ethics committees of the German Federal States where all study centers were located (Bayerische Landesärztekammer (protocol code 13,023, Approval Date: 27 March 2013 and 14 February 2014 (rectification of documents, study protocol, consent form, etc.)).

## Results

The basic descriptive parameters of the study population (age and sex distribution) are shown in Table 1.

**Table 1:** Linear regression analyses relating serum urate concentrations (exposure variable) to pulse wave velocity (outcome variable), adjusting for relevant confounders (whole study sample, *n* = 70,649; complete model: *r*^2^ = 0.524). *SD* standard deviation, *IQR* interquartile range

Variable	Mean ± SD	Median (IQR)	Regression coefficient (confidence interval)	*p* value
Arterial pulse wave velocity (m/s)	10.72 ± 1.97	10.5 (9.3–11.9)		
Serum urate (µmol/l)	286 ± 80	279 (227–338)	0.0012 (0.0010; 0.0013)	< 0.001
Sex (male, female)	Male: 47.1% Female: 52.9%		− 0.132 (− 0.160; − 0.103)	< 0.001
Age (years)	51.4 ± 12.4	53 (44–62)	0.072 (0.071; 0.073)	< 0.001
BMI (kg/m^2^)	26.6 ± 4.8	25.9 (23.1–29.1)	− 0.033 (− 0.035; − 0.030)	< 0.001
Smoking (never, past, present)	Never: 45.6% Past: 33.9% Present: 20.5%		Past: − 0.083 (− 0.106; − 0.059)	< 0.001
			Present: − 0.104 (− 0.132; − 0.077)	< 0.001
Alcohol intake (g/day)	10.8 ± 16.8	4.67 (1.293–13.395)	0.0024 (0.0018; 0.0031)	< 0.001
HbA1c (mmol/mol)	36.7 ± 6.5	36 (33–39)	0.018 (0.016; 0.02)	< 0.001
HDL cholesterol (mmol/l)	1.56 ± 0.44	1.5 (1.24–1.82)	− 0.061 (− 0.089; − 0.033)	< 0.001
LDL cholesterol (mmol/l)	3.36 ± 0.92	3.31 (2.71–3.94)	0.045 (0.033; 0.057)	< 0.001
Serum creatinine (µmol/l)	72.7 ± 15.9	71 (62–81)	− 0.002 (− 0.002; − 0.001)	< 0.001
Physical activity (MET-min/week)	7657 ± 9541	4080 (1600–10,080)	6.17·10^−7^ (− 4.44·10^−7^; 1.68·10^−6^)	0.255
Systolic blood pressure (mmHg)	128.7 ± 16.7	127 (117–139)	0.045 (0.045; 0.046)	< 0.001
Anti-hypertensive drugs (yes/no)	Yes: 26.1% No: 73.9%		0.128 (0.100; 0.155)	< 0.001
Lipid-lowering drugs (yes/no)	Yes: 9.4% No: 90.6%		0.055 (0.016; 0.093)	0.006
Urate-lowering drugs (yes/no)	Yes: 2.3% No: 97.7%		0.290 (0.220; 0.361)	< 0.001

### Association of PWV with serum uric acid

**Fig. 1:**
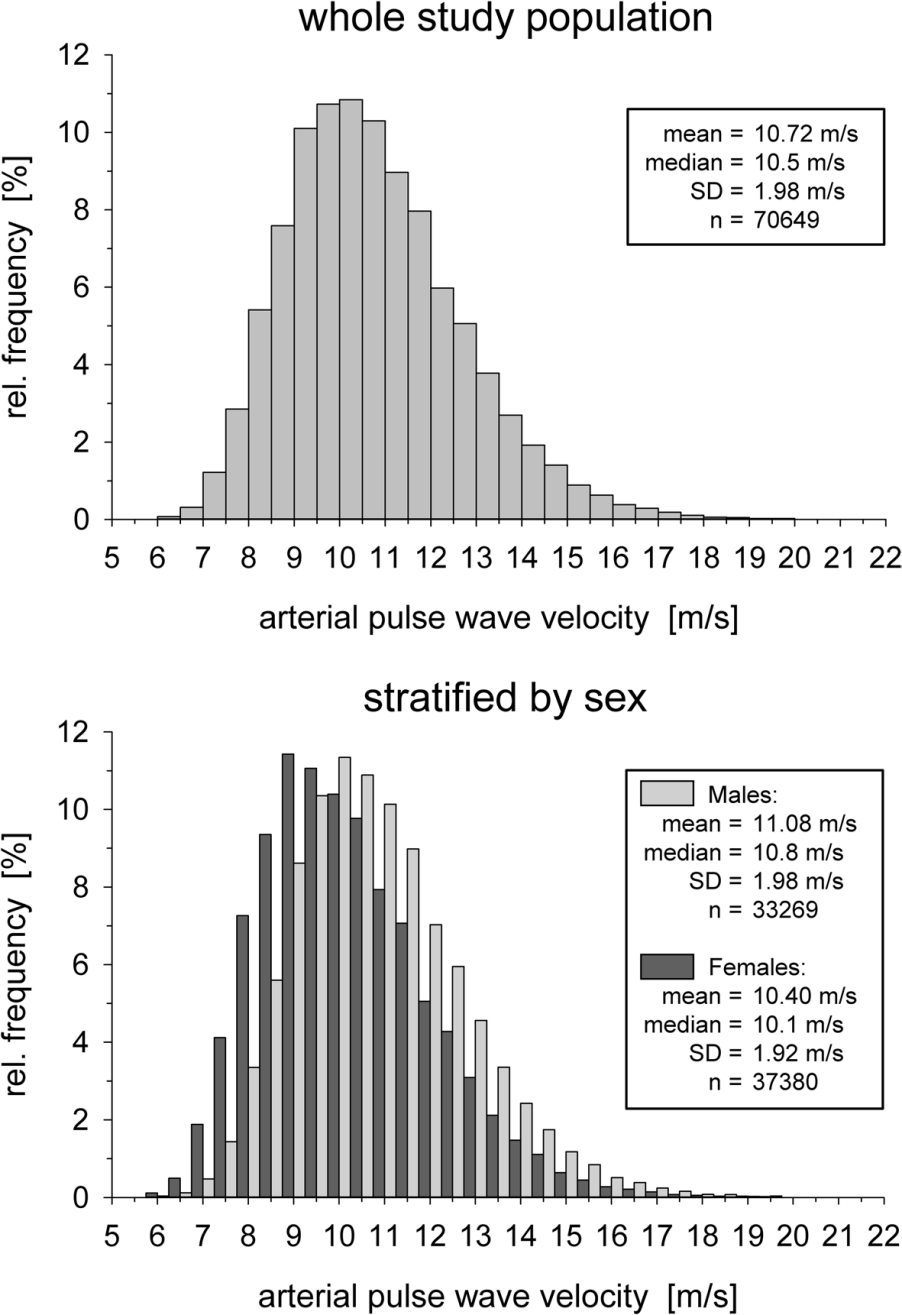
Frequency distribution of the arterial pulse wave velocity in the study population and stratified by sex

Figure 1 shows the distribution of arterial pulse wave velocity (PWV) in the total study sample (upper panel) and separately for female and male adults (lower panel). As expected, the distribution is shifted to slightly higher values for male as compared to female adults. Overall, the values obtained with the Vascular Explorer™ [56] are in the expected range for the general adult population [59, 60].

Table 1 summarizes the descriptive statistics and the regression analysis for PWV with serum uric acid concentration and the other above named independent variables for the entire study sample. An initial scatter plot analysis showed no indication for a deviation from linearity. The machine learning approach (see"Methods"section) supported this. There is a positive association of serum urate with PWV as dependent variable even after adjustment for confounding variables. The distribution of the standardized residuals of this model is shown in Additional file 1: Fig. S03 and fulfills the assumption of a normal distribution. We also checked the regression model for any multicollinearity within the variables by calculating the variance inflation factor (VIF). The value of this factor laid between 1.013 and 1.952 for all model variables, indicating that collinearity seems to play only a minor role in the actual analysis. Similar low VIF values were found for all other regression models calculated in this study.

The same analysis was performed for different age groups (< 31 years, 31–50 years, > 50 years). The results (Additional file 1: Tables S03a–S03c) clearly show in all age groups a strong impact of the serum urate level on the arterial pulse wave velocity, indicating that the role of urate for vascular stiffness seems to be not the result of increasing age. Not surprisingly for some other variables (e.g., anti-hypertensive medication or smoking history) the impact on PWV was age-group-dependent.

### Positive association of serum uric acid with PWV in the physiological concentration range

Next, we repeated the analyses, restricting the sample to those study participants with serum uric acid values in the “normal” concentration range (male: ]180, 420] µmol/l; female: ]140, 360] µmol/l) used by most laboratories [5] and obtained almost the same effect estimate for serum urate as in the overall sample (Table 2), indicating that the association between serum uric acid and PWV persists. Thus, the association of uric acid is not restricted to hyperuricemia.

**Table 2:** Linear regression analyses relating serum urate concentrations (exposure variable) to pulse wave velocity (outcome variable), adjusting for relevant confounders restricted to individuals with normal serum urate levels (male: ]180, 420] µmol/l; female: ]140, 360] µmol/l), *n* = 64,095; complete model: *r*^2^ = 0.526. *SD* standard deviation, *IQR* interquartile range

Variable	Mean ± SD	Median (IQR)	Regression coefficient (confidence interval)	*p* value
Arterial pulse wave velocity (m/s)	10.67 ± 1.97	10.4 (9.3–11.8)		
Serum urate (µmol/l)	276 ± 64	273 (226–324)	0.0012 (0.0009; 0.0014)	< 0.001
Sex (male, female)	Male: 46.0% Female: 54.0%		− 0.112 (− 0.143; − 0.082)	< 0.001
Age (years)	51.0 ± 12.4	52 (44–61)	0.071 (0.070; 0.072)	< 0.001
BMI (kg/m^2^)	26.3 ± 4.6	25.6 (23–28.8)	− 0.033 (− 0.036; − 0.031)	< 0.001
Smoking (never, past, actual)	Never: 46.2% Past: 33.1% Actual: 20.7%		Past: − 0.088 (− 0.112; − 0.063)	< 0.001
			Actual: − 0.094 (− 0.123; − 0.066)	< 0.001
Alcohol intake (g/day)	10.4 ± 16.2	4.596 (1.284–12.871)	0.002 (0.0013; 0.0027)	< 0.001
HbA1c (mmol/mol)	36.6 ± 6.3	36 (33–39)	0.019 (0.017; 0.021)	< 0.001
HDL cholesterol (mmol/l)	1.57 ± 0.44	1.52 (1.26–1.84)	− 0.066 (− 0.096; − 0.037)	< 0.001
LDL cholesterol (mmol/l)	3.35 ± 0.91	3.29 (2.7–3.92)	0.046 (0.033; 0.059)	< 0.001
Serum creatinine (µmol/l)	71.9 ± 14.6	71 (62–80)	− 0.001 (− 0.002; − 0.001)	< 0.001
Physical activity (MET-min/week)	7623 ± 9521	4080 (1600–10,080)	4.24·10^−7^ (− 6.84·10^−7^; 1.53·10^−6^)	0.453
Systolic blood pressure (mmHg)	128.4 ± 16.6	127 (117–138)	0.046 (0.045; 0.046)	< 0.001
Anti-hypertensive drugs (yes/no)	Yes: 23.8% No: 76.2%		0.138 (0.11; 0.167)	< 0.001
Lipid-lowering drugs (yes/no)	Yes: 8.8% No: 91.2%		0.081 (0.04; 0.123)	< 0.001
Urate-lowering drugs (yes/no)	Yes: 2.1% No: 97.9%		0.280 (0.204; 0.357)	< 0.001

The sub-group analysis showed the same effect of urate on PWV in individuals with “normal” concentration in all age groups (Additional file 1: Tables S04a–S04c).

### Sex-dependent positive association of serum uric acid with PWV arterial stiffness

Tables 3 and 4 show the results of the regression analysis separately for female and male adults including either all participants or only the ones with “normal” serum uric acid levels. The slope of the regression is steeper for female adults compared to male adults. To analyze whether sex indeed leads to differences in the relation between serum urate level and pulse wave velocity, an additional regression model was calculated in which an interaction term of sex and urate level was included. The results showed a highly significant (*p* < 0.001) impact of this interaction with PWV which indicates that the different slopes of urate level and PWV between males and females are statistically significant.

**Table 3:** Regression analysis; female population, *n* = 37,380; complete model: *r*^2^ = 0.552; male population, *n* = 33,269; complete model: *r*^2^ = 0.468. *SD* standard deviation, *IQR* interquartile range

Variable	Female				Male			
	**Mean ± SD**	**Median (IQR)**	***b* (95%CI)**	***p* value**	**Mean ± SD**	**Median (IQR)**	***b* (95%CI)**	***p* value**
Arterial pulse wave velocity (m/s)	10.40 ± 1.92	10.1 (9.0–11.5)			11.08 ± 1.98	10.8 (9.7–12.2)		
Serum urate (µmol/l)	245 ± 64	238 (201–280)	0.0012 (0.0010; 0.0015)	< 0.001	332 ± 70	327 (284–375)	0.0012 (0.0009; 0.0014)	< 0.001
Age (years)	50.9 ± 12.4	52 (44–61)	0.068 (0.067; 0.070)	< 0.001	52.0 ± 12.4	53 (45–62)	0.075 (0.073; 0.076)	< 0.001
BMI (kg/m^2^)	25.9 ± 5.1	24.8 (22.2–28.6)	− 0.042 (− 0.045; − 0.039)	< 0.001	27.3 ± 4.3	26.7 (24.4–29.6)	− 0.019 (− 0.023; − 0.015)	< 0.001
Smoking (never, past, actual)	Never: 50.5% Past: 30.4% Actual: 19.1%		Past: − 0.125 (− 0.155; − 0.095)	< 0.001	Never: 40.1% Past: 37.8% Actual: 22.1%		Past: − 0.046 (− 0.082; − 0.009)	0.014
			Actual: − 0.125 (− 0.161; − 0.089)	< 0.001			Actual: − 0.073 (− 0.115; − 0.031)	< 0.001
Alcohol intake (g/day)	7.3 ± 12.5	3.116 (0.8345–9.071)	− 4.24·10^−5^ (− 0.0011; 0.0010)	0.938	14.8 ± 19.9	8.071 (2.525–19.271)	0.0034 (0.0026; 0.0042)	< 0.001
HbA1c (mmol/mol)	36.3 ± 5.7	36 (33–39)	0.015 (0.013; 0.018)	< 0.001	37.3 ± 7.2	36 (33–39)	0.019 (0.016; 0.021)	< 0.001
HDL cholesterol (mmol/l)	1.74 ± 0.43	1.7 (1.44–2.00)	− 0.032 (− 0.066; 0.002)	0.061	1.36 ± 0.35	1.31 (1.10–1.55)	− 0.099 (− 0.147; − 0.050)	< 0.001
LDL cholesterol (mmol/l)	3.33 ± 0.93	3.25 (2.66–3.90)	0.066 (0.050; 0.082)	< 0.001	3.40 ± 0.91	3.38 (2.77–3.98)	0.031 (0.013; 0.050)	< 0.001
Serum creatinine (µmol/l)	65.1 ± 11.9	64 (58–71)	− 0.003 (− 0.004; − 0.002)	< 0.001	81.2 ± 15.6	80 (72–88)	− 0.001 (− 0.002; 0.000)	0.130
Physical activity (MET-min/week)	7292 ± 9037	4000 (1560–9640)	1.64·10^−6^ (1.94·10^−7^; 3.1·10^−6^)	0.026	8068 ± 10,062	4200 (1600–10,680)	− 2.39·10^−7^ (− 1.8·10^−6^; 1.3·10^−6^)	0.763
Systolic blood pressure (mmHg)	124.9 ± 16.9	122 (113–135)	0.046 (0.046; 0.047)	< 0.001	133.1 ± 15.4	131 (123–142)	0.045 (0.044; 0.046)	< 0.001
Anti-hypertensive drugs (yes/no)	Yes: 22.9% No: 77.1%		0.143 (0.107; 0.180)	< 0.001	Yes: 29.6% No: 70.4%		0.101 (0.059; 0.142)	< 0.001
Lipid-lowering drugs (yes/no)	Yes: 6.6% No: 93.4%		0.129 (0.073; 0.186)	< 0.001	Yes: 12.6% No: 87.4%		− 0.016 (− 0.070; 0.039)	0.571
Urate-lowering drugs (yes/no)	Yes: 0.6% No: 99.4%		0.158 (− 0.013; 0.329)	0.070	Yes: 4.1% No: 95.9%		0.251 (0.169; 0.333)	< 0.001

**Table 4:** Regression analysis of female persons with normal serum urate levels (]140, 360] µmol/l; *n* = 34,595; complete model: *r*^2^ = 0.556) and of male persons with normal serum urate levels (]180, 420] µmol/l; *n* = 29,500; complete model: *r*^2^ = 0.468). *SD* standard deviation, *IQR* interquartile range

Variable	Female				Male			
	**Mean ± SD**	**Median (IQR)**	***b* (95%CI)**	***p* value**	**Mean ± SD**	**Median (IQR)**	***b* (95%CI)**	***p* value**
Arterial pulse wave velocity (m/s)	10.36 ± 1.90	10.1 (9.0–11.5)			11.02 ± 1.98	10.8 (9.7–12.1)		
Serum urate (µmol/l)	239 ± 50	236 (202–274)	0.0014 (0.0010; 0.0017)	< 0.001	318 ± 53	319 (280–358)	0.0011 (0.0008; 0.0015)	< 0.001
Age (years)	50.6 ± 12.4	52 (43–61)	0.069 (0.067; 0.070)	< 0.001	51.6 ± 12.4	53 (44–62)	0.074 (0.072; 0.075)	< 0.001
BMI (kg/m^2^)	25.7 ± 4.9	24.7 (22.1–28.2)	− 0.043 (− 0.046; − 0.040)	< 0.001	27.0 ± 4.2	26.5 (24.2–29.2)	− 0.019 (− 0.023; − 0.014)	< 0.001
Smoking (never, past, actual)	Never: 50.6% Past: 30.2% Actual: 19.2%		Past: − 0.134 (− 0.166; − 0.103)	< 0.001	Never: 41.1% Past: 36.6% Actual: 22.4%		Past: − 0.041 (− 0.08; − 0.003)	0.036
			Actual: − 0.119 (− 0.155; − 0.082)	< 0.001			Actual: − 0.058 (− 0.103; − 0.014)	0.010
Alcohol intake (g/day)	7.3 ± 12.4	3.161 (0.871–9.104)	− 0.0003 (− 0.0014; 0.0008)	0.566	14.1 ± 19.3	7.7685 (2.4–18.2968)	0.003 (0.0021; 0.0039)	< 0.001
HbA1c (mmol/mol)	36.1 ± 5.5	36 (33–38)	0.015 (0.012; 0.018)	< 0.001	37.1 ± 7.2	36 (33–39)	0.020 (0.017; 0.023)	< 0.001
HDL cholesterol (mmol/l)	1.75 ± 0.43	1.70 (1.45–2.00)	− 0.048 (− 0.083; − 0.014)	0.006	1.37 ± 0.35	1.32 (1.11–1.57)	− 0.088 (− 0.139; − 0.036)	< 0.001
LDL cholesterol (mmol/l)	3.31 ± 0.92	3.23 (2.66–3.88)	0.063 (0.047; 0.080)	< 0.001	3.39 ± 0.90	3.36 (2.76–3.96)	0.034 (0.014; 0.054)	< 0.001
Serum creatinine (µmol/l)	64.8 ± 11.1	64 (58–71)	− 0.003 (− 0.004; − 0.001)	< 0.001	80.3 ± 13.8	79 (72–87)	− 0.001 (− 0.002; 0.001)	0.307
Physical activity (MET-min/week)	7256 ± 9001	3960 (1560–9600)	1.19·10^−6^ (− 3.06·10^−7^; 2.69·10^−6^)	0.119	8052 ± 10,082	4080 (1600–10,560)	− 2.75·10^−7^ (− 1.92·10^−6^; 1.37·10^−6^)	0.743
Systolic blood pressure (mmHg)	124.6 ± 16.8	122 (113–134)	0.047 (0.046; 0.048)	< 0.001	132.8 ± 15.3	131 (122–141)	0.045 (0.043; 0.046)	< 0.001
Anti-hypertensive drugs (yes/no)	Yes: 20.9% No: 79.1%		0.152 (0.114; 0.189)	< 0.001	Yes: 27.1% No: 72.9%		0.113 (0.069; 0.157)	< 0.001
Lipid-lowering drugs (yes/no)	Yes: 6.0% No: 94.0%		0.151 (0.090; 0.211)	< 0.001	Yes: 12.0% No: 88.0%		0.011 (− 0.048; 0.07)	0.705
Urate-lowering drugs (yes/no)	Yes: 0.5% No: 99.5%		0.125 (− 0.067; 0.316)	0.202	Yes: 3.9% No: 96.1%		0.239 (0.150; 0.328)	< 0.001

The differences in the regression coefficients in Table 4 reflect the conclusion of the interaction term of sex and urate level, showing a significant impact of this interaction. Thus, the results indicate that the different slopes of urate level and PWV are statistically significant and that the impact of urate on vascular stiffness is stronger for female adults in the “normal” serum concentration range.

**Fig. 2:**
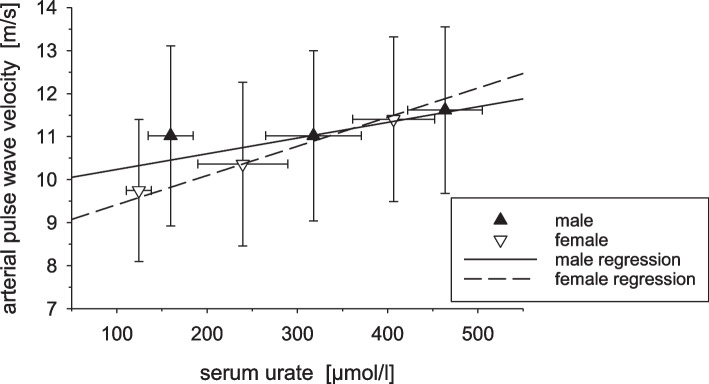
Association of pulse wave velocity and serum urate level for male (*n* = 33,803) and female (*n* = 37,953) persons. Serum urate was classified in low (male: ≤ 180 µmol/l, female: ≤ 140 µmol/l), normal (male: 180 < – ≤ 420 µmol/l, female: 140 < – ≤ 360 µmol/l), and high (male: > 420 µmol/l, female: > 360 µmol/l) values. The regression lines were calculated from all individual data in the respective sex group (not adjusted for other covariates). For the presentation, the data were grouped and the means ± SD of serum urate and PWV for each group are shown

Figure 2 compares directly the dependency of PWV and serum urate for female and male adults.

### Quantification of feature importance by machine learning

**Fig. 3:**
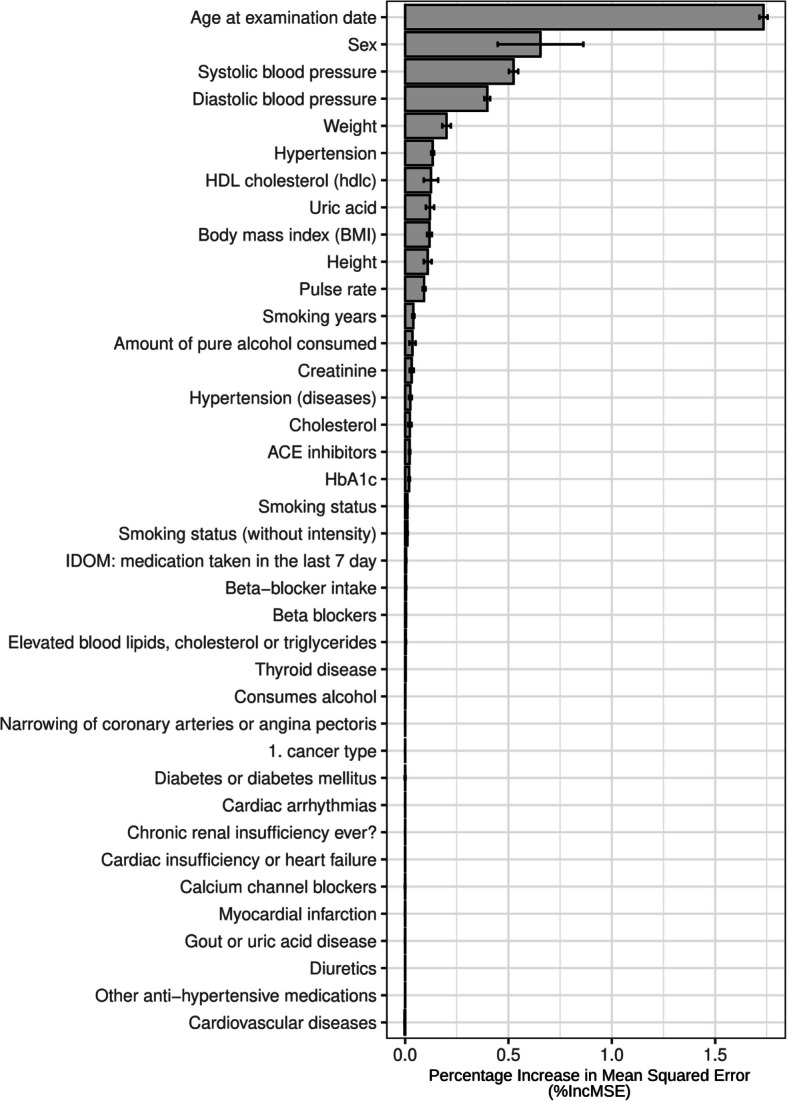
Feature importance measured by mean %IncMSE using random forests in a tenfold CV scheme; values are reported by mean and standard deviation. Hypertension = measured systolic ≥ 140 or diastolic blood pressure ≥ 90; hypertension (disease) = self-reported diagnosis of hypertension; self-reported cardiovascular diseases = intermittent claudication or circulatory disorders in the legs, also known as intermittent claudication or arterial occlusive disease; 1 st cancer type = entity of the first diagnosed cancer if cancer has ever been diagnosed

Figure 3 shows the feature importance of the 38 variables used to predict PWV measured by the percent increase (%IncMSE) of mean squared error (MSE) resulting from training random forests in a tenfold cross-validation (CV) scheme; a larger value indicates stronger relevance for predicting PWV. Concerning redundancy, systolic and diastolic blood pressure are influenced physiologically to varying degrees by different hemodynamic, cardiac, and vascular factors and are therefore not redundant. Likewise, hypertension is not redundant with the blood pressure parameters, since in one case the physiological blood pressure range is included and in the other case only the pathological range is taken into account. Likewise, body height, body weight, and BMI are related but not redundant [61–63].

The ten highest ranking variables are age at time of examination, sex, systolic blood pressure, diastolic blood pressure, weight, high blood pressure (hypertension), HDL cholesterol measurement, urate measurement (uric acid), BMI, and body size (height). Uric acid ranges among the top eight variables.

## Discussion

In humans, uric acid is the end product of purine metabolism (metabolism of DNA, RNA, or nucleotides) that requires renal excretion to maintain homeostasis. An increase in uric acid formation or impaired elimination can lead to gout and urolithiasis due to crystallization [5]. Although it is a metabolic end product, physiological serum concentrations in the range of ~ 200 to ~ 400 µmol/l are maintained at steady state. The reason behind that is probably the blood pressure-increasing or -maintaining effect of uric acid, which represented an evolutionary advantage during the transition to terrestrial life when NaCl supply was critical [5, 19]. This effect is probably based on the vascular impact of uric acid, which can promote phenotypic changes of vascular smooth muscle cells and endothelial cells, thereby leading to vascular remodeling and ultimately higher blood pressure [64–67]. Recent studies demonstrate an increase in oxidative stress, a decrease in NO availability, activation of the renin–angiotensin–aldosterone system (RAAS), and vascular inflammatory processes as underlying molecular mechanisms [68–70].

Nowadays, NaCl supply is abundant and there is overconsumption, making this impact detrimental and contributing to vascular pathophysiology. Accordingly, several studies indicate that increased uric acid serum concentrations are associated with cardiovascular diseases and vascular end-organ damage [2, 29, 46, 71]. Thus, it is conceivable that uric acid promotes vascular dysfunction and vascular remodeling and thereby arterial hypertension. However, this hypothesis is unproven for humans, and it cannot be excluded that uric acid promotes primarily hypertension, thereby leading to vascular damage. In addition, pathophysiologically relevant uric acid concentration ranges as well as their sex differences are unclear.

Our present analyses show the positive association of serum uric acid concentrations with PWV, as a measure of vascular stiffness in a large and population-based cohort of the German adult population. This association was observed across the entire spectrum of serum urate levels and not restricted to individuals with hyperuricemia and persisted after correction for relevant confounders, including blood pressure, age, smoking, obesity, blood glucose, Hba1c, or cholesterol levels. Of note, the strength of the association was identical (same regression coefficient) in the entire sample and in the subsample with “normal” serum urate levels [5]. The result of machine learning approach, performed as a proof of principle, confirmed the regression analysis and identified uric acid as independent variable associated with vascular stiffness. Of note, linear regression analysis resulted in negative regression coefficients for smoking related to PWV. This finding is surprising but highly significant and should be analyzed further in subsequent studies. However, it is important to note that the effect size is small.

As PWV and vascular stiffness are well established indicators for vascular remodeling in humans [72], our data describe a possible missing mechanistic link between the known pathological impact of uric acid on vascular cells and its documented role as a risk factor for vascular end-organ damage in humans.

In view of these data, combined with the putative evolutionary role of uric acid, the question about the appropriateness of existing cut-off values for normal urate concentrations arises. Possibly, there are no “normal” uric acid concentrations under our current environmental conditions or the “ideal” (i.e., healthiest) concentration ranges are much lower [5]. This issue needs further consideration in future studies. Another aspect to be considered is the question whether treatment with urate-lowering drugs (uricostatics or uricosurics) lead to classification of persons under risk in the group of persons with “normal” urate level. In our analyzed cohort of 64,095 individuals with urate levels in the physiological range, 1340 (= 2.1%) received urate-lowering drugs. To take this aspect into account, the analyses were performed with the information of urate-lowering drugs as a confounding variable. As shown in Table 2, the plasma urate level is still correlated with the PWV even when adjusted for urate-lowering drugs in this group. This holds also true for the sex-specific analyses (Table 4). However, in our analysis we do not have any information of how long these drugs were taken in the past. Therefore, the general question whether a successful treatment prevents long-term changes in vascular stiffness or even with treatment a cellular mechanism is still acting cannot be answered.

The sub-group analysis of different age groups revealed that the urate effect on PWV (even in individuals with “normal” concentration) occurs in young as well as in aged persons (Additional file 1: Tables S03 and S04). The role of other variables analyzed occurred to be age-dependent. For instance, the smoking history plays a role only in elderly people but not in younger. Here, the duration of the smoking history in the past could be relevant.

In view of the well-known sex dependence of vascular health, we tested whether the association between serum uric acid and vascular stiffness differs between female and male adults. Surprisingly, vascular stiffness increased in a more pronounced manner with serum uric acid in females than in male adults. The slope of the regression line in female adults of Fig. 2 was approximately 6.8 m·l·s^−1^·mmol^−1^ for females and 3.7 m·l·s^−1^·mmol^−1^ for males; i.e., velocity increases by 0.68 m/s or 0.37 m/s for every 100 µmol/l of serum uric acid. Analyzing the age-dependent increase in PWV, we obtained regression slopes of 0.099 m·s^−1^·year^−1^ and 0.094 m·s^−1^·year^−1^ for females and males, respectively. These values agree well with data from literature [59, 60, 73]. Comparison of the uric acid-dependent increase in vascular stiffness with the age-dependent increases in vascular stiffness indicates that an increase of serum uric acid by 100 µmol/l corresponds to an increase of vascular stiffness equivalent to ~ 7 years of increase in age in female and ~ 4 years in males. Of course, this is only a first estimate and not corrected for confounding factors. Yet, also a smaller impact of uric acid could be functional relevant. In this simple regression analysis between urate and PWV, the value of *r*^2^ is 0.057. Separate analyses for females and males result in a *r*^2^ value for women of 0.053 and for men of 0.015. In a combined model that only takes urate and gender into account (related to PWV), *r*^2^ is 0.059. Adding an interaction term of urate and gender to this model results in a *r*^2^ of 0.062.

In analogy to the simplistic estimation of the impact of urate level and age on PWV (shown above), a rough but illustrative calculation of the impact of other variables in relation to age can be done. For instance, an increase in the BMI by 5 results in a higher PWV which is equivalent to an increase in age of ~ 4 years. For other variables, it would be ΔHbA1c by + 10 mmol/mol ≃ + 9 years, ΔLDL cholesterol by + 1 mmol/l ≃ + 5 years, or Δsystolic RR by + 10 mmHg ≃ + 7 years.

Although surprising on the first view, our results may help to explain the stronger impact of urate and the more beneficial effect of urate-lowering interventions on cardiovascular variables in female as compared to male individuals [29, 39, 40]. These results emphasize the importance of female-male comparative biomedical research [53, 74] in order to evaluate health risks and appropriate health care strategies adequately for our societies. The dimension sex adds another level of complexity to the issue of “normal” uric acid concentrations and the possible benefits of interventions. Furthermore, our data add to a recent study on the association of gout (crystal arthropathy due to hyperuricemia) with cardiovascular diseases, showing that the impact is stronger in women than in men [41]. According to our results, vascular remodeling may be an underlying pathomechanism. Furthermore, the present results suggest that the higher risk for women is not restricted to pathological high uric. Our results are supported by several other studies, showing a stronger association between uremic acid serum concentrations and vascular function in women than in men [28, 75, 76]. Genetic or sex steroid dependent differences in vascular ROS homeostasis, NO production, RAAS activation, and immune response may account for these alterations in the response to uric acid [77–79].

As an additional plausibility check and sensitivity analysis, which may identify and quantify associated factors regarding PWV, we applied a random forest-based machine learning approach. Furthermore, we wanted to gain information concerning the possible suitability of this approach using our extensive data set and our hypothesis driven research question as a use case. The machine learning approach confirmed uric acid as an important factor associated with PWV and ranked it within the top eight variables. The other top ranked variables are well known and validated regarding vascular remodeling and PWV alterations. Thus, random forest-based machine learning approach could be a valid AI tool for the identification and quantification of strength of risk factors and should be further validated.

Of course, our study has strengths and limitations. The large number of participants, the large number of parameters determined, and the possibility to compare male and female participants are without doubt strengths. On the other hand, in such a large multi-center study the question may arise whether the measured values (laboratory values, PWV measurements) are comparable between different study centers. All participating laboratories are accredited, certified laboratories that fulfill the guidelines of the German Medical Association for the quality assurance of laboratory medical examinations. In particular, the stability of the uric acid values over the study period and potential differences between the laboratories were analyzed. Only neglectable minor discrepancies were observed. Another critical aspect of the study results from inclusion of only complete datasets in the analysis. From the initial 101,556 participants, only 70,649 (70.0%) were included in the statistical analysis (Additional file 1: Fig. S01). The question arises whether the exclusion of 30% of the participants may lead to a selection bias. Additional file 1: Table S05 shows the description of the variables in the included and excluded cohort. The comparison does not show marked differences between the groups. Especially PVW and urate level are almost identical. In addition, the other variables are “only” confounders for which the statistical model is adjusted. Therefore, a systematic bias by exclusion of participants can be ruled out.

A general limitation of our cross-sectional study is that it does not allow conclusions regarding causality. This aspect has to be addressed in detail at different experimental levels, from experimental studies with primary vascular cells of both sexes to interventional studies in humans. In addition, a follow-up with a longitudinal design should be performed. Furthermore, one has to be aware that not all possible confounders for the relationship of uric acid with arterial stiffness (e.g., additional lifestyle factors, environmental exposures, diet quality) can be considered in a single study and potentially relevant missing confounders should be addressed in the follow-up studies.

## Conclusions

The relationship of PWV and serum uric acid unveiled by our study can help to close the knowledge gap between the previously described impact of urate on vasculature and the epidemiological evidence for urate as risk factor for vascular end-organ damage. The results also represent a framework to explain the positive effect of strong serum uric acid lowering interventions on cardiovascular events, as well as the sex-dependent efficacy of these interventions. Future studies have to address the sex difference mechanistically at the cellular level by comparing the impact of urate on vascular smooth muscle cells and endothelial cells from female and male donors. Furthermore, intervention studies should be performed to confirm the conclusions at the systemic level in humans and to quantify more precisely the relative contribution of uric acid on vascular stiffness.

## Supplementary Information

Additional file 1. Flow chart of included participants and additional linear regression analysis. Fig. S01: Flow chart numbers of included participants. Table. S03a: Linear regression analyses relating serum urate concentrationsto pulse wave velocity, adjusting for relevant confounders for individuals with age <31 years. Table. S03b: Linear regression analyses relating serum urate concentrationsto pulse wave velocity, adjusting for relevant confounders for individuals with age 31–-50 years. Table. S03c: Linear regression analyses relating serum urate concentrationsto pulse wave velocity, adjusting for relevant confounders for individuals with age >50 years. Table. S04a: Linear regression analyses relating serum urate concentrationsto pulse wave velocity, adjusting for relevant confounders restricted to individuals with normal serum urate levels (male: ]180, 420] μmol/l; female: ]140, 360] μmol/l) and age <31 years, n = 5707; complete model: r^2^ = 0.250. SD = standard deviation, IQR = interquartile range. Table. S04b: Linear regression analyses relating serum urate concentrationsto pulse wave velocity, adjusting for relevant confounders restricted to individuals with normal serum urate levels (male: ]180, 420] μmol/l; female: ]140, 360] μmol/l) and age 31–-50 years, n = 22,784; complete model: r^2^ = 0.316. SD = standard deviation, IQR = interquartile range. Table. S04c: Linear regression analyses relating serum urate concentrationsto pulse wave velocity, adjusting for relevant confounders restricted to individuals with normal serum urate levels (male: ]180, 420] μmol/l; female: ]140, 360] μmol/l) and age >50 years, n = 35,604; complete model: r^2^ = 0.356. SD = standard deviation, IQR = interquartile range. Table. S05: Description of the model variables in persons included in the analysisand in the group excluded due to incomplete data sets.. Fig. S03: Distributions of the standardized residuals of the regression models described in Tables 1,2,3 and 4

Additional file 2. Additional information on the machine learning procedure. Table. S01: Percent increaseof mean squared errorfor each of the 38 variables used to predict PWV with random forests in a 10-fold CV scheme. Hypertension = measured systolic blood pressure ≥ 140 or diastolic blood pressure ≥ 90; hypertension= self-reported hypertension; cardiovascular diseases = intermittent claudication or circulatory disorders in the legs, also known as intermittent claudication or arterial occlusive disease; 1st cancer type = entity of the first diagnosed cancer if cancer has ever been diagnosed. Fig. S02: Boxplots for each individual fold. These results indicate how well the 10 individual random forestsdid adapt to the non-linear regression task and to what extent results varied across all ten folds. Red lines indicate a threshold of +5% or −-5%, respectively. Table. S02: Descriptive statistics for each individual fold. These results equal the data visualized in Fig. S02. A mean error of 1% means that a given test data sample, the random forest did on average deviate from the actual target value by 1% of that value

## Data Availability

Original data can be requested from the NAKO transfer office (transfer-nako@med.uni-greifswald.de; https://transfer.nako.de/transfer/contact). NAKO ensures that the same dataset used in this study will be made available via the transfer office in order to comply with good scientific practice.

## Abbreviations

ATC: Anatomical therapeutic chemical
BMI: Body mass index
CV: Cross-validation
IQR: Interquartile range
MSE: Mean squared error
NAKO: German National Cohort
%IncMSE: Percent increase of MSE
PWV: Pulse wave velocity
RAAS: Renin-angiotensin-aldosterone system
RF: Random forest
SD: Standard deviation
URAT1: Urate transporter 1
VIF: Variance inflation factor
