# Supplementary material for: Physiological serum uric acid concentrations correlate with arterial stiffness in a sex-dependent manner
Source: BMC Med. 2025 Jul 1;23:356. doi: 10.1186/s12916-025-04195-8 (PMC12211650; doi:10.1186/s12916-025-04195-8)
Supplement: Supplementary file 1 — Additional file 1. Flow chart of included participants and additional linear regression analysis. Fig. S01: Flow chart numbers of included participants. Table. S03a: Linear regression analyses relating serum urate concentrationsto pulse wave velocity, adjusting for relevant confounders for individuals with age <31 years. Table. S03b: Linear regression analyses relating serum urate concentrationsto pulse wave velocity, adjusting for relevant confounders for individuals with age 31–-50 years. Table. S03c: Linear regression analyses relating serum urate concentrationsto pulse wave velocity, adjusting for relevant confounders for individuals with age >50 years. Table. S04a: Linear regression analyses relating serum urate concentrationsto pulse wave velocity, adjusting for relevant confounders restricted to individuals with normal serum urate levels (male: ]180, 420] μmol/l; female: ]140, 360] μmol/l) and age <31 years, n = 5707; complete model: r2 = 0.250. SD = standard deviation, IQR = interquartile range. Table. S04b: Linear regression analyses relating serum urate concentrationsto pulse wave velocity, adjusting for relevant confounders restricted to individuals with normal serum urate levels (male: ]180, 420] μmol/l; female: ]140, 360] μmol/l) and age 31–-50 years, n = 22,784; complete model: r2 = 0.316. SD = standard deviation, IQR = interquartile range. Table. S04c: Linear regression analyses relating serum urate concentrationsto pulse wave velocity, adjusting for relevant confounders restricted to individuals with normal serum urate levels (male: ]180, 420] μmol/l; female: ]140, 360] μmol/l) and age >50 years, n = 35,604; complete model: r2 = 0.356. SD = standard deviation, IQR = interquartile range. Table. S05: Description of the model variables in persons included in the analysisand in the group excluded due to incomplete data sets.. Fig. S03: Distributions of the standardized residuals of the regression models described in Tables 1,2,3 and 4 [file 12916_2025_4195_MOESM1_ESM.pdf]

# Physiological serum uric acid concentrations correlate with arterial stiffness in a sex-dependent manner.

O. Thews, T. Schmid, A. Kluttig, A. Wienke, M. Zinkhan, W. Ahrens, T. Bärnighausen, H. Brenner, S. Castell, B. Lange, W. Lieb, K.H. Greiser, M. Dörr, L. Krist, S.N. Willich, V. Harth, N. Obi, M. Leitzmann, A. Peters, B. Schmidt, M. B. Schulze, H. Völzke, M. Nauck, S. Zylla, A. Hannemann, T. Pischon, I.M. Velásquez, M. Girndt, C. Grossmann, M. Gekle

**Additional file 1**

**Fig. S01:** Flow chart numbers of included participants

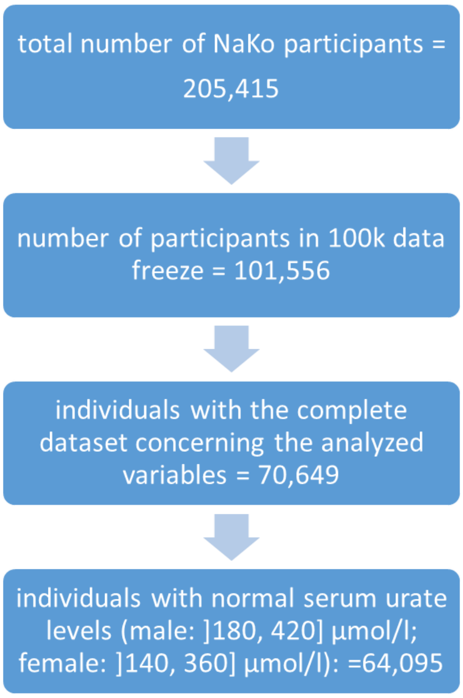

**Tab. S03a:** Linear regression analyses relating serum urate concentrations (exposure variable) to pulse wave velocity (outcome variable), adjusting for relevant confounders for individuals **with age <31 years** (n=6063; complete model: $r^2$=0.254. SD = standard deviation, IQR = interquartile range).

| Variable | Mean ± SD | Median (IQR) | Regression coefficient (confidence interval) | p-value |
|---|---|---|---|---|
| Arterial pulse wave velocity (m/s) | 8.64 ± 0.97 | 8.6 (8.0 - 9.3) | | |
| Serum urate (µmol/l) | 266 ± 74 | 258 (212 - 314) | 0.0008 (0.0004; 0.0012) | <0.001 |
| Sex (male, female) | male: 43.6% female: 56.4% | | -0.146 (-0.214; -0.078) | <0.001 |
| Age (years) | 26.2 ± 2.7 | 26 (24 - 28) | 0.060 (0.052; 0.068) | <0.001 |
| BMI (kg/m$^2$) | 24.1 ± 4.3 | 23.4 (21.2 - 26.1) | -0.021 (-0.027; -0.016) | <0.001 |
| Smoking (never, past, present) | never: 57.9% past: 16.3% present: 25.8% | | past: 0.004 (-0.056; 0.065) present: 0.04 (-0.012; 0.093) | 0.885 0.131 |
| Alcohol intake (g/day) | 8.2 ± 13.1 | 4.44 (1.50 - 9.70) | -0.0005 (-0.0022; 0.0012) | 0.575 |
| HbA1c (mmol/mol) | 32.7 ± 3.8 | 33 (30 - 35) | 0.003 (-0.002; 0.009) | 0.263 |
| HDL cholesterol (mmol/l) | 1.57 ± 0.41 | 1.53 (1.28 - 1.82) | 0.038 (-0.023; 0.098) | 0.224 |
| LDL cholesterol (mmol/l) | 2.67 ± 0.73 | 2.59 (2.15 - 3.12) | 0.093 (0.063; 0.124) | <0.001 |
| Serum creatinine (µmol/l) | 70.6 ± 15.5 | 69 (61 - 79) | -0.002 (-0.004; 0.000) | 0.011 |
| Physical activity (MET-min/week) | 7165 ± 9156 | 3720 (1680 - 8840) | $-6.94 \cdot 10^{-7}$ ($-3.04 \cdot 10^{-6}$; $1.65 \cdot 10^{-6}$) | 0.562 |
| Systolic blood pressure (mmHg) | 120.4 ± 12.4 | 120 (112 - 128) | 0.032 (0.030; 0.034) | <0.001 |
| Anti-hypertensive drugs (yes/no) | yes: 1.9% no: 98.1% | | 0.222 (0.065; 0.380) | 0.006 |
| Lipid-lowering drugs (yes/no) | yes: 0.5% no: 99.5% | | -0.084 (-0.388; 0.220) | 0.589 |
| Urate-lowering drugs (yes/no) | yes: 0.1% no: 99.9% | | 0.807 (0.065; 1.548) | 0.033 |

**Tab. S03b:** Linear regression analyses relating serum urate concentrations (exposure variable) to pulse wave velocity (outcome variable), adjusting for relevant confounders for individuals **with age 31-50 years** (n=24523; complete model: $r^2$=0.323. SD = standard deviation, IQR = interquartile range).

| Variable | Mean ± SD | Median (IQR) | Regression coefficient (confidence interval) | p-value |
|---|---|---|---|---|
| Arterial pulse wave velocity (m/s) | 9.79 ± 1.43 | 9.7 (8.9 - 10.6) | | |
| Serum urate (µmol/l) | 271 ± 78 | 262 (213 - 321) | 0.0009 (0.0006; 0.0012) | <0.001 |
| Sex (male, female) | male: 45.6% female: 54.4% | | -0.137 (-0.182; -0.093) | <0.001 |
| Age (years) | 43.1 ± 5.6 | 44 (39 - 48) | 0.052 (0.050; 0.055) | <0.001 |
| BMI (kg/m$^2$) | 25.9 ± 4.8 | 25.1 (22.6 - 28.3) | -0.019 (-0.022; -0.015) | <0.001 |
| Smoking (never, past, present) | never: 46.8% past: 29.2% present: 23.9% | | past: -0.028 (-0.064; 0.007) present: 0.014 (-0.025; 0.052) | 0.112 0.479 |
| Alcohol intake (g/day) | 9.1 ± 14.7 | 4.06 (1.18 - 11.09) | 0.0008 (-0.0003; 0.0019) | 0.136 |
| HbA1c (mmol/mol) | 34.9 ± 5.2 | 35 (32 - 37) | 0.017 (0.014; 0.02) | <0.001 |
| HDL cholesterol (mmol/l) | 1.54 ± 0.42 | 1.5 (1.23 - 1.79) | -0.068 (-0.111; -0.024) | 0.002 |
| LDL cholesterol (mmol/l) | 3.22 ± 0.86 | 3.14 (2.61 - 3.75) | 0.068(0.05; 0.087) | <0.001 |
| Serum creatinine (µmol/l) | 71.6 ± 14.4 | 70 (62 - 80) | -0.002 (-0.003; -0.001) | 0.001 |
| Physical activity (MET-min/week) | 7124 ± 9613 | 3320 (1240 - 9120) | $7.09 \cdot 10^{-7}$ ($-8.40 \cdot 10^{-7}$; $2.26 \cdot 10^{-6}$) | 0.370 |
| Systolic blood pressure (mmHg) | 124.0 ± 14.4 | 123 (114 - 133) | 0.044 (0.043; 0.045) | <0.001 |
| Anti-hypertensive drugs (yes/no) | yes: 10.5% no: 89.5% | | 0.181 (0.130; 0.233) | <0.001 |
| Lipid-lowering drugs (yes/no) | yes: 2.5% no: 97.5% | | 0.186 (0.089; 0.284) | <0.001 |
| Urate-lowering drugs (yes/no) | yes: 0.6% no: 99.4% | | 0.204 (0.006; 0.402) | 0.044 |

**Tab. S03c:** Linear regression analyses relating serum urate concentrations (exposure variable) to pulse wave velocity (outcome variable), adjusting for relevant confounders for individuals **with age >50 years** (n=40063; complete model: $r^2$=0.350. SD = standard deviation, IQR = interquartile range).

| Variable | Mean ± SD | Median (IQR) | Regression coefficient (confidence interval) | p-value |
|---|---|---|---|---|
| Arterial pulse wave velocity (m/s) | 11.61 ± 1.87 | 11.4 (10.3 - 12.7) | | |
| Serum urate (µmol/l) | 298 ± 80 | 292 (240 - 349) | 0.0011 (0.0009; 0.0014) | <0.001 |
| Sex (male, female) | male: 48.5% female: 51.5% | | -0.165 (-0.205; -0.125) | <0.001 |
| Age (years) | 60.3 ± 5.8 | 60 (55 - 65) | 0.093 (0.090; 0.095) | <0.001 |
| BMI (kg/m$^2$) | 27.3 ± 4.7 | 26.7 (24.0 - 29.9) | -0.039 (-0.042; -0.035) | <0.001 |
| Smoking (never, past, present) | never: 43.0% past: 39.4% present: 17.6% | | past: -0.113 (-0.146; -0.079) present: -0.175 (-0.210; -0.132) | <0.001 <0.001 |
| Alcohol intake (g/day) | 12.2 ± 18.4 | 5.35 (1.33 - 15.93) | 0.0032 (0.0023; 0.0041) | <0.001 |
| HbA1c (mmol/mol) | 38.5 ± 6.9 | 37 (35 - 40) | 0.018 (0.016; 0.021) | <0.001 |
| HDL cholesterol (mmol/l) | 1.57 ± 0.45 | 1.5 (1.24 - 1.84) | -0.066 (-0.106; -0.025) | 0.001 |
| LDL cholesterol (mmol/l) | 3.56 ± 0.92 | 3.52 (2.92 - 4.14) | 0.048 (0.031; 0.066) | <0.001 |
| Serum creatinine (µmol/l) | 73.6 ± 16.8 | 72 (63 - 82) | -0.001 (-0.002; 0.000) | 0.029 |
| Physical activity (MET-min/week) | 8057 ± 9535 | 4720 (1840 - 10800) | $1.46 \cdot 10^{-7}$ ($-1.41 \cdot 10^{-6}$; $1.70 \cdot 10^{-6}$) | 0.853 |
| Systolic blood pressure (mmHg) | 132.9 ± 17.4 | 131 (121 - 143) | 0.046 (0.045; 0.047) | <0.001 |
| Anti-hypertensive drugs (yes/no) | yes: 39.2% no: 60.8% | | 0.078 (0.044; 0.113) | <0.001 |
| Lipid-lowering drugs (yes/no) | yes: 15.0% no: 85.0% | | 0.001 (-0.046; 0.048) | 0.965 |
| Urate-lowering drugs (yes/no) | yes: 3.6% no: 96.4% | | 0.264 (0.181; 0.347) | <0.001 |

**Tab. S04a:** Linear regression analyses relating serum urate concentrations (exposure variable) to pulse wave velocity (outcome variable), adjusting for relevant confounders restricted to individuals with normal serum urate levels (male: ]180, 420] µmol/l; female: ]140, 360] µmol/l) **and age <31 years**, n=5707; complete model: $r^2$=0.250. SD = standard deviation, IQR = interquartile range.

| Variable | Mean ± SD | Median (IQR) | Regression coefficient (confidence interval) | p-value |
|---|---|---|---|---|
| Arterial pulse wave velocity (m/s) | 8.62 ± 0.96 | 8.6 (8.0 - 9.2) | | |
| Serum urate (µmol/l) | 263 ± 63 | 257 (214 - 309) | 0.0007 (0.0002; 0.0012) | 0.003 |
| Sex (male, female) | male: 42.8% female: 57.2% | | -0.173 (-0.247; -0.100) | <0.001 |
| Age (years) | 26.2 ± 2.7 | 26 (24 - 28) | 0.059 (0.051; 0.067) | <0.001 |
| BMI (kg/m$^2$) | 24 ± 4.2 | 23.3 (21.1 – 26.0) | -0.022 (-0.028; -0.016) | <0.001 |
| Smoking (never, past, present) | never: 58.1% past: 16.2% present: 25.7% | | past: 0.015 (-0.048; 0.077) present: 0.052 (-0.001; 0.106) | 0.644 0.057 |
| Alcohol intake (g/day) | 8 ± 13 | 4.4 (1.48 - 9.42) | 0.0003 (-0.0021; 0.0014) | 0.723 |
| HbA1c (mmol/mol) | 32.7 ± 3.8 | 33 (30 - 35) | 0.002 (-0.004; 0.008) | 0.445 |
| HDL cholesterol (mmol/l) | 1.58 ± 0.41 | 1.53( 1.29 - 1.83) | 0.029 (-0.033; 0.091) | 0.361 |
| LDL cholesterol (mmol/l) | 2.66 ± 0.73 | 2.59 (2.15 - 3.1) | 0.096 (0.064; 0.127) | <0.001 |
| Serum creatinine (µmol/l) | 70.4 ± 12.9 | 69 (61 - 79) | -0.003 (-0.006; -0.001) | 0.003 |
| Physical activity (MET-min/week) | 7107 ± 9052 | 3720 (1680 - 8720) | $-1.01 \cdot 10^{-6}$ ($-3.44 \cdot 10^{-6}$; $1.42 \cdot 10^{-6}$) | 0.414 |
| Systolic blood pressure (mmHg) | 120.1 ± 12.3 | 119 (111 - 128) | 0.032 (0.030; 0.034) | <0.001 |
| Anti-hypertensive drugs (yes/no) | yes: 1.7% no: 98.3% | | 0.175 (0.006; 0.344) | 0.043 |
| Lipid-lowering drugs (yes/no) | yes: 0.5% no: 99.5% | | -0.156( -0.469; 0.157) | 0.329 |
| Urate-lowering drugs (yes/no) | yes: 0.1% no: 99.9% | | 0.992 (0.165; 1.818) | 0.019 |

**Tab. S04b:** Linear regression analyses relating serum urate concentrations (exposure variable) to pulse wave velocity (outcome variable), adjusting for relevant confounders restricted to individuals with normal serum urate levels (male: ]180, 420] µmol/l; female: ]140, 360] µmol/l) **and age 31-50 years**, n=22784; complete model: $r^2$=0.316. SD = standard deviation, IQR = interquartile range.

| Variable | Mean ± SD | Median (IQR) | Regression coefficient (confidence interval) | p-value |
|---|---|---|---|---|
| Arterial pulse wave velocity (m/s) | 9.76 ± 1.43 | 9.6 (8.9 - 10.5) | | |
| Serum urate (µmol/l) | 266 ± 65 | 260 (214 - 313) | 0.0007 (0.0004; 0.0011) | <0.001 |
| Sex (male, female) | male: 44.8% female: 55.2% | | -0.149 (-0.198; -0.101) | <0.001 |
| Age (years) | 43.1 ± 5.6 | 44 (39 - 48) | 0.053 (0.050; 0.055) | <0.001 |
| BMI (kg/m$^2$) | 25.8 ± 4.6 | 25.0 (22.6 – 28.0) | -0.019 (-0.023; -0.015) | <0.001 |
| Smoking (never, past, present) | never: 47.1% past: 29.0% present: 23.9% | | past: -0.024 (-0.061; 0.013) present: 0.026 (-0.014; 0.066) | 0.1.97 0.200 |
| Alcohol intake (g/day) | 8.9 ± 14.1 | 4.00 (1.18 - 10.87) | $-2.77\cdot10^{-5}$ (-0.0011; 0.0011) | 0.963 |
| HbA1c (mmol/mol) | 34.9 ± 5.1 | 35 (32 - 37) | 0.018 (0.014; 0.021) | <0.001 |
| HDL cholesterol (mmol/l) | 1.55 ± 0.42 | 1.5 (1.24 - 1.80) | -0.058 (-0.103; -0.013) | 0.012 |
| LDL cholesterol (mmol/l) | 3.2 ± 0.85 | 3.13 (2.61 - 3.72) | 0.067 (0.047; 0.086) | <0.001 |
| Serum creatinine (µmol/l) | 71.3 ± 13.1 | 70 (62 - 80) | -0.002 (-0.004; -0.001) | 0.008 |
| Physical activity (MET-min/week) | 7074 ± 9561 | 3280 (1240 - 9000) | $7.05\cdot10^{-7}$ ($-9.21\cdot10^{-7}$; $2.33\cdot10^{-6}$) | 0.395 |
| Systolic blood pressure (mmHg) | 123.8 ± 14.3 | 123 (114 - 132) | 0.044 (0.043; 0.045) | <0.001 |
| Anti-hypertensive drugs (yes/no) | yes: 9.8% no: 90.2% | | 0.194 (0.139; 0.249) | <0.001 |
| Lipid-lowering drugs (yes/no) | yes: 2.4% no: 97.6% | | 0.197 (0.093; 0.302) | <0.001 |
| Urate-lowering drugs (yes/no) | yes: 0.5% no: 99.5% | | 0.203 (-0.01; 0.417) | 0.062 |

**Tab. S04c:** Linear regression analyses relating serum urate concentrations (exposure variable) to pulse wave velocity (outcome variable), adjusting for relevant confounders restricted to individuals with normal serum urate levels (male: ]180, 420] µmol/l; female: ]140, 360] µmol/l) **and age >50 years**, n=35604; complete model: $r^2$=0.356. SD = standard deviation, IQR = interquartile range.

| **Variable** | **Mean ± SD** | **Median (IQR)** | **Regression coefficient (confidence interval)** | **p-value** |
|---|---|---|---|---|
| Arterial pulse wave velocity (m/s) | 11.57 ± 1.87 | 11.4 (10.3 - 12.6) | | |
| Serum urate (µmol/l) | 284 ± 63 | 283 (237 - 331) | 0.0012 (0.0008; 0.0015) | <0.001 |
| Sex (male, female) | male: 47.3% female: 52.7% | | -0.130 (-0.174; -0.086) | <0.001 |
| Age (years) | 60.1 ± 5.8 | 60 (55 - 65) | 0.094 (0.091; 0.096) | <0.001 |
| BMI (kg/m$^2$) | 27 ± 4.6 | 26.4 (23.8 - 29.5) | -0.039 (-0.043; -0.035) | <0.001 |
| Smoking (never, past, present) | never: 43.7% past: 38.5% present: 17.8% | | past: -0.124 (-0.159; -0.089) present: -0.170 (-0.215; -0.125) | <0.001 <0.001 |
| Alcohol intake (g/day) | 11.8 ± 17.8 | 5.21 (1.33 - 14.99) | 0.0029 (0.0019; 0.0038) | <0.001 |
| HbA1c (mmol/mol) | 38.3 ± 6.8 | 37 (35 - 40) | 0.020 (0.017; 0.022) | <0.001 |
| HDL cholesterol (mmol/l) | 1.59 ± 0.45 | 1.53 (1.26 - 1.86) | -0.078 (-0.121; -0.036) | <0.001 |
| LDL cholesterol (mmol/l) | 3.55 ± 0.91 | 3.51 (2.92 - 4.12) | 0.050 (0.031; 0.068) | <0.001 |
| Serum creatinine (µmol/l) | 72.6 ± 15.7 | 71 (62 - 81) | -0.001 (-0.002; 0.000) | 0.137 |
| Physical activity (MET-min/week) | 8056 ± 9548 | 4680 (1840 - 10800) | $-2.60\cdot10^{-7}$ ($-1.90\cdot10^{-6}$; $1.39\cdot10^{-6}$) | 0.756 |
| Systolic blood pressure (mmHg) | 132.7 ± 17.3 | 131 (121 - 143) | 0.046 (0.045; 0.047) | <0.001 |
| Anti-hypertensive drugs (yes/no) | yes: 36.3% no: 63.7% | | 0.088 (0.051; 0.124) | <0.001 |
| Lipid-lowering drugs (yes/no) | yes: 14.2% no: 85.8% | | 0.026 (-0.024; 0.076) | 0.310 |
| Urate-lowering drugs (yes/no) | yes: 3.4% no: 96.6% | | 0.249 (0.159; 0.340) | <0.001 |

**Tab. S05:** Description of the model variables in persons included in the analysis (n=70649) and in the group excluded due to incomplete data sets. (SD = standard deviation, IQR = interquartile range).

| | Included cohort | | Excluded cohort | | |
|---|---|---|---|---|---|
| **Variable** | **Mean ± SD** | **Median (IQR)** | **Mean ± SD** | **Median (IQR)** | **n** |
| Arterial pulse wave velocity (m/s) | 10.72 ± 1.97 | 10.5 (9.3 - 11.9) | 10.93 ± 2.04 | 10.7 (9.5 - 12.1) | 19,638 |
| Serum urate (µmol/l) | 286 ± 80 | 279 (227 - 338) | 286 ± 81 | 280 (226 - 336.8) | 25,468 |
| Sex (male, female) | male: 47.1% female: 52.9% | | male: 45.0% female: 55.0% | | 30,907 |
| Age (years) | 51.4 ± 12.4 | 53 (44 - 62) | 53.4 ± 12.2 | 55 (46 - 64) | 30,907 |
| BMI (kg/m$^2$) | 26.6 ± 4.8 | 25.9 (23.1 - 29.1) | 27.5 ± 5.7 | 26.5 (23.5 - 30.3) | 27,138 |
| Smoking (never, past, present) | never: 45.6% past: 33.9% present: 20.5% | | never: 43.6% past: 35.4% present: 21.0% | | 25,618 |
| Alcohol intake (g/day) | 10.8 ± 16.8 | 4.67 (1.29 - 13.40) | 10.8 ± 18.1 | 4.25 (0.95 - 13.30) | 24,842 |
| HbA1c (mmol/mol) | 36.7 ± 6.5 | 36 (33 - 39) | 37.8 ± 7.7 | 37 (34 - 40) | 25,082 |
| HDL cholesterol (mmol/l) | 1.56 ± 0.44 | 1.50 (1.24 - 1.82) | 1.54 ± 0.45 | 1.48 (1.22 - 1.81) | 24,810 |
| LDL cholesterol (mmol/l) | 3.36 ± 0.92 | 3.31 (2.71 - 3.94) | 3.34 ± 0.94 | 3.29 (2.68 - 3.95) | 20,288 |
| Serum creatinine (µmol/l) | 72.7 ± 15.9 | 71 (62 - 81) | 73.2 ± 21 | 71 (62 - 82) | 25,550 |
| Physical activity (MET-min/week) | 7657 ± 9541 | 4080 (1600 - 10080) | 6198 ± 9009 | 2800 (240 - 8120) | 24,784 |
| Systolic blood pressure (mmHg) | 128.7 ± 16.7 | 127 (117 - 139) | 129.8 ± 17.9 | 128 (117 - 140) | 30,663 |
| Anti-hypertensive drugs (yes/no) | yes: 26.1% no: 9.4% | | yes: 32.4% no: 67.6% | | 28,468 |
| Lipid-lowering drugs (yes/no) | yes: 26.1% no: 90.6% | | yes: 11.9% no: 88.1% | | 30,907 |
| Urate-lowering drugs (yes/no) | yes: 2.3% no: 97.7% | | yes: 3.1% no: 96.9% | | 30,907 |

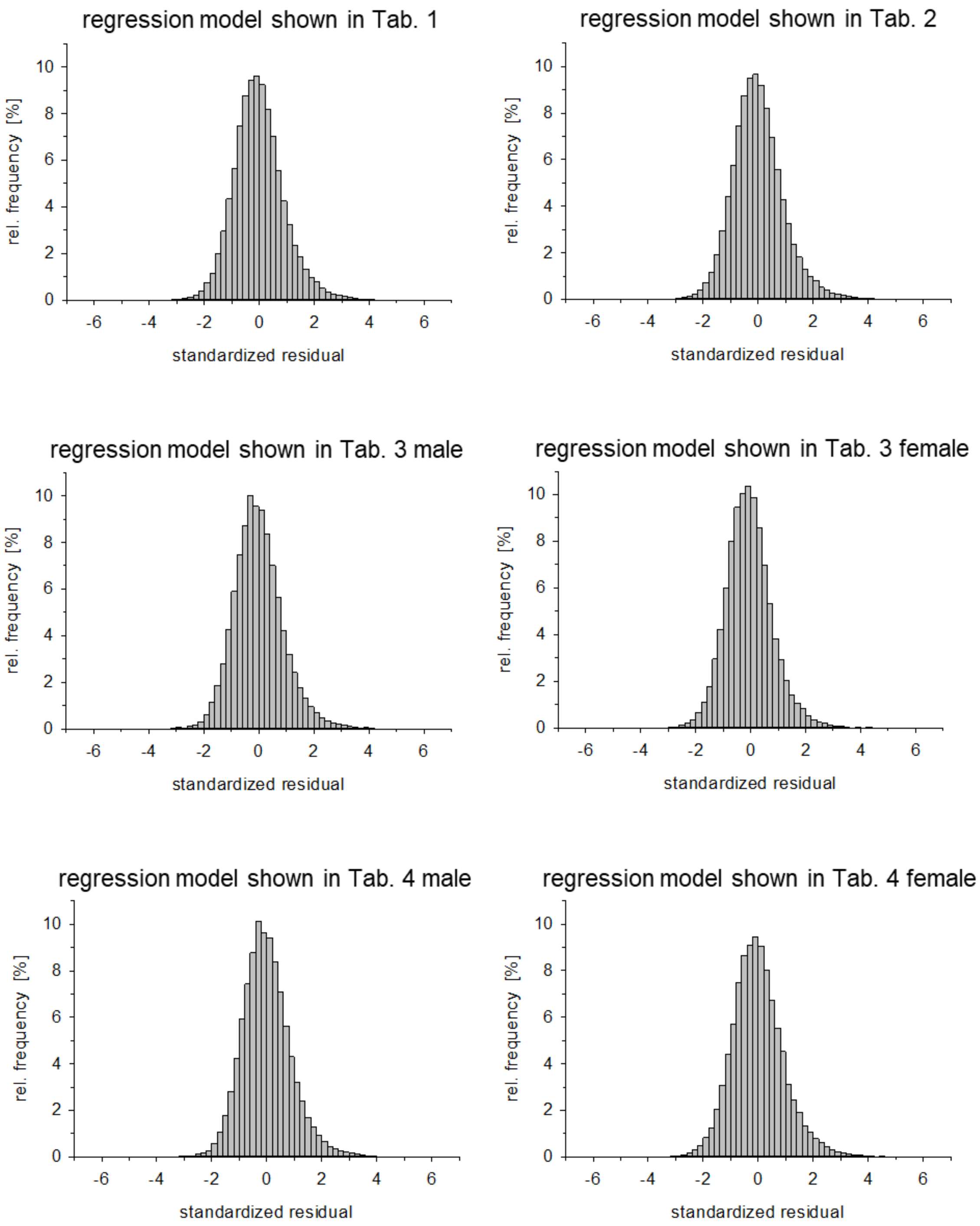


**Fig. S03:** Distributions of the standardized residuals of the regression models described in Tabs. 1-4.
