# Supplementary material for: Physiological serum uric acid concentrations correlate with arterial stiffness in a sex-dependent manner
Source: BMC Med. 2025 Jul 1;23:356. doi: 10.1186/s12916-025-04195-8 (PMC12211650; doi:10.1186/s12916-025-04195-8)
Supplement: Supplementary file 2 — Additional file 2. Additional information on the machine learning procedure. Table. S01: Percent increaseof mean squared errorfor each of the 38 variables used to predict PWV with random forests in a 10-fold CV scheme. Hypertension = measured systolic blood pressure ≥ 140 or diastolic blood pressure ≥ 90; hypertension= self-reported hypertension; cardiovascular diseases = intermittent claudication or circulatory disorders in the legs, also known as intermittent claudication or arterial occlusive disease; 1st cancer type = entity of the first diagnosed cancer if cancer has ever been diagnosed. Fig. S02: Boxplots for each individual fold. These results indicate how well the 10 individual random forestsdid adapt to the non-linear regression task and to what extent results varied across all ten folds. Red lines indicate a threshold of +5% or −-5%, respectively. Table. S02: Descriptive statistics for each individual fold. These results equal the data visualized in Fig. S02. A mean error of 1% means that a given test data sample, the random forest did on average deviate from the actual target value by 1% of that value [file 12916_2025_4195_MOESM2_ESM.pdf]

Physiological serum uric acid concentrations correlate with arterial stiffness in a sex-dependent manner.

O. Thews, T. Schmid, A. Kluttig, A. Wienke, M. Zinkhan, W. Ahrens, T. Bärnighausen, H. Brenner, S. Castell, B. Lange, W. Lieb, K.H. Greiser, M. Dörr, L. Krist, S.N. Willich, V. Harth, N. Obi, M. Leitzmann, A. Peters, B. Schmidt, M. B. Schulze, H. Völzke, M. Nauck, S. Zylla, A. Hannemann, T. Pischon, I.M. Velásquez, M. Girndt, C. Grossmann, M. Gekle

Additional file 2:

Feature Importance Assessment by Machine Learning

(I) FEATURE IMPORTANCE BY VARIABLE

To assess the performance of our Random Forests-based predictor for Pulse Wave Velocity (PWV), we employed a 10-fold Cross-Validation (CV) scheme. For each of the 38 variables used as input, we calculated the percentage increase in MSE (%IncMSE). Tab. S04 reports mean and standard deviation value across all ten folds of the 10-fold CV scheme.

The %IncMSE metric measures how much each input variable contributes to the overall improvement or reduction in prediction error. A higher %IncMSE value for a particular input variable indicates that it has a significant impact on reducing the mean squared error between predicted and actual values. In other words, features with high %IncMSE are those whose removal would lead to an increase in prediction errors, making them more important contributors to the model's performance. The R package “randomForest”, which we use, employs uses this metric as part of its feature importance calculation, providing a way to rank variables based on their relative impact on the model's predictions.

Tab. S01: Percent increase (%IncMSE) of mean squared error (MSE) for each of the 38 variables used to predict PWV with random forests in a 10-fold CV scheme. Hypertension = measured systolic >=140 or diastolic blood pressure >= 90; hypertension (disease) = self-reported hypertension; cardiovascular diseases = intermittent claudication or circulatory disorders in the legs, also known as intermittent claudication or arterial occlusive disease; 1st cancer type = entity of the first diagnosed cancer if cancer has ever been diagnosed.

Variable	*Mean*	*Standard Deviation*
Age at examination date	1.734	0.020
Sex	0.655	0.207
Systolic blood pressure	0.524	0.022
Diastolic blood pressure	0.397	0.014
Weight	0.200	0.021
Hypertension	0.133	0.007
HDL cholesterol (hdlc)	0.125	0.035
Uric acid	0.120	0.020
Body mass index (BMI)	0.118	0.012
Height	0.109	0.019
Pulse rate	0.092	0.007
Smoking years	0.040	0.005
Amount of pure alcohol consumed	0.035	0.015
Creatinine	0.032	0.010
Hypertension (diseases)	0.025	0.007
Cholesterol	0.023	0.008
ACE inhibitors	0.021	0.003
HbA1c	0.019	0.004
Smoking status	0.011	0.001
Smoking status (without intensity)	0.011	0.002
IDOM: medication taken in the last 7 day	0.005	0.001
Beta-blocker intake	0.004	0.001
Beta blockers	0.004	0.001
Elevated blood lipids, cholesterol or triglycerides	0.003	0.001
Thyroid disease	0.002	0.000
Consumes alcohol	0.001	0.000
Calcium channel blockers	0.000	0.001
Narrowing of coronary arteries or angina pectoris	0.000	0.000
1. cancer type	0.000	0.000
Diabetes or diabetes mellitus	0.000	0.002
Cardiac arrhythmias	0.000	0.000
Chronic renal insufficiency ever?	0.000	0.000
Cardiac insufficiency or heart failure	0.000	0.000
Myocardial infarction	0.000	0.001
Gout or uric acid disease	0.000	0.001
Diuretics	0.000	0.000
Other anti-hypertensive medications	0.000	0.000
Cardiovascular diseases	-0.003	0.000

(II) TRAINING OF RANDOM FORESTS

Used Implementation: R package „random Forest“ [1]

Training Scheme: 10-fold Cross-Validation

Training Parameters:

Parameter	*Value*
Number of trees	500
Number of variables randomly sampled as candidates at each split	22
Minimum size of terminal nodes	1
Sampling of cases for each tree	With replacement
Size of the sample drawn to grow each tree	Full training data
Priors of the classes for classification	Equal priors

Results for Individual Folds of 10-fold Cross-Validation with Random Forests

In order to assess the success of training the ten individual Random Forest regressors of the 10-fold Cross-Validation scheme, we report Percentage Error (predicted-target)/target*100) as error measure for both Fig. S02 and Tab. S02.

Note that Fig. S02 is a visualization of Tab. S02, where both refer to folds 1 to 10 of the 10-fold Cross Validation scheme. %IncMSE values achieved with these ten individual Random Forest models (not shown) are the basis for the 10-fold CV feature importance results reported in Tab. S01.

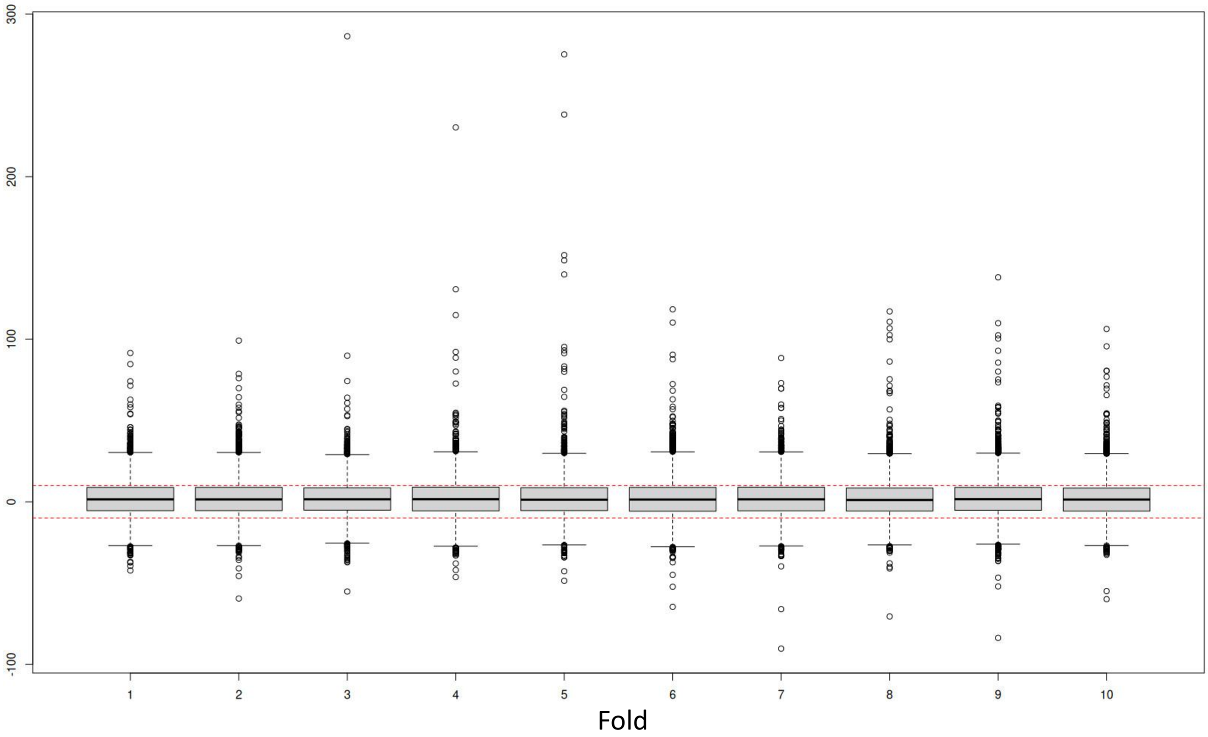

Fig. S02: Boxplots for each individual fold (metric: percentage error; test data: n=7449). These results indicate how well the 10 individual random forests (trained on an individual data fold) did adapt to the non-linear regression task and to what extent results varied across all ten folds. Red lines indicate a threshold of +5% or -5%, respectively.

---

1 https://cran.r-project.org/web/packages/randomForest/index.html

Tab. S02: Descriptive statistics for each individual fold (metric: percentage error; test data: n=7449). These results equal the data visualized in Fig S02. A mean error of 1% means that a given test data sample, the random forest did on average deviate from the actual target value by 1% of that value.

Fold	*Min*	*1st Qu*	*Median*	*Mean*	*3rd Qu*	*Max*
1	-42.26	-5.482	1.51	1.908	8.86	91.542
2	-59.479	-5.434	1.479	2.017	8.887	99.165
3	-55.187	-5.129	1.551	1.977	8.584	286.381
4	-46.301	-5.611	1.603	2.064	9.009	230.329
5	-48.565	-5.422	1.308	2.016	8.672	275.243
6	-64.592	-5.783	1.403	1.932	8.851	118.397
7	-90.305	-5.555	1.513	1.959	8.976	88.471
8	-70.526	-5.696	1.121	1.741	8.467	117.13
9	-83.709	-5.229	1.629	2.198	8.86	138.08
10	-59.877	-5.708	1.401	1.69	8.424	106.333

(III) EXCLUDED VARIABLES REMOVED DUE TO MISSING VALUES

a) Due to missing values or which are directly correlated with other parameters in the model

Variables with more than 5,000 missing entries (approximately 5% of samples) were removed in order to eliminate features with excessive missing data that could introduce bias or noise:

- Age at which the consumption of alcoholic beverages was quit (for former consumers)
- Age at initial diagnosis of angina pectoris
- Age at initial diagnosis of cardiac arrhythmias
- Age at initial diagnosis of arterial occlusive disease in the legs
- Age at initial diagnosis of cardiac insufficiency or heart failure
- Age at initial diagnosis of hypertension
- Age at diagnosis of first heart attack
- Age at diagnosis of second heart attack
- Age at initial diagnosis Elevated blood lipids or cholesterol and/or triglyceride
- Age at initial diagnosis of diabetes mellitus
- Duration of treatment with insulin
- Duration of treatment with pills
- Age at initial diagnosis of gout or a uric acid disease
- Age at initial diagnosis of thyroid disease
- Adjusted body fat percentage measured in % and entered automatically or manually
- Quality Phase angle (measured, adjusted)
- Age of the proband at the end of the smoking career
- Are WHO recommendations on physical activity met?
- Age at initial cancer diagnosis in years
- Type of first cancer, edited from d_an_ca1_f/m and free text data from d_an_ca1_f/m_t
- Age at second cancer diagnosis in years
- Type of second cancer, edited from d_an_ca2_f/m and free text data from d_an_ca2_f/m_t
- Age at third cancer diagnosis in years
- Type of third cancer, edited from d_an_ca3_f/m and free text data from d_an_ca3_f/m_t
- Age at fourth cancer diagnosis in years
- Type of fourth cancer, edited from d_an_ca4_f/m and free text data from d_an_ca4_f/m_t
- Age at heart valve surgery
- Age at coronary artery balloon dilatation (PTCA) with/without stent implantation

- Age at cardiac bypass surgery
- Age at pacemaker implantation
- Age at balloon dilatation of the leg arteries with/without stent implantation
- Age at bypass surgeries on the leg arteries
- Age at carotid artery surgery
- Age at complete or partial removal of the thyroid/parathyroid gland
- Smoking intensity as number of packyears (smoking duration [years] x lifetime_cigs/20)
- Highest educational qualification, allocated according to ISCED 97 level
- Age of the proband at the beginning of the smoking career
- Average number of cigars, cigarillos or pipes smoked per day (ever to date)
- Average number of cigarettes smoked per day (ever to date)
- Have you received medical treatment for angina pectoris within the last 12 months?
- Have you received medical treatment for cardiac arrhythmias within the last 12 months?
- Have you received medical treatment for arterial occlusive disease in the legs within the last 12 months?
- Have you received medical treatment for heart failure or cardiac insufficiency within the last 12 months?
- Have you received medical treatment for hypertension within the last 12 months?
- How were you treated back then? (1. heart attack outpatient)
- How were you treated back then? (last heart attack)
- How many heart attacks have you had?
- Have you received medical treatment for elevated blood lipids or cholesterol and/or triglycerides in the last 12 months?
- Was this diagnosis first detected during pregnancy?
- How are you currently being treated? (e.g., injected insulin and insulin pumps)
- Have you received medical treatment for gout or uric acid disease in the last 12 months?
- Have you received medical treatment for thyroid disease in the last 12 months?
- Treatment with thyroid hormones (Thyroid gland Type of treatment)
- Treatment with radioiodine therapy (Thyroid gland Type of treatment)
- Treatment with radiation in the neck area (Thyroid gland Type of treatment)
- Treatment with thyroid surgery (Thyroid gland Type of treatment)
- Treatment with iodine tablets (Thyroid gland Type of treatment)
- Other treatment (Thyroid gland Type of treatment)
- Impaired renal function (year, age) (d_an_neph1_a)
- Impaired renal function (year, age) (d_an_neph1_j)
- Have you ever been treated with dialysis?
- Do you often have swollen legs in the evening?
- Do you have to get up regularly at night to urinate?
- How often is that usually per night?
- Do you experience shortness of breath or a feeling of faintness or both during physical exertion?
- At what level of exertion do shortness of breath or feelings of faintness occur?
- Do you normally sleep with your upper body elevated?
- How high does your upper body approximately lie?
- Glucose (gluk): Measured value or error code (6 digits)
- C-reactive protein, highly sensitive (hscrp): Measured value or error code (6 digits)
- LDL cholesterol (ldlc): Measured value or error code (6 digits)
- Urea (urea): Measured value or error code (6 digits)
- Leukocytes (wbc): Measured value or error code (6 digits)

b) Vascular Explorer variables

- Heart rate (heart frequency)
- Distance centre upper arm cuff - jugulum

- Distance jugulum - symphysis
- Distance jugulum - middle ankle cuff
- statistically calculated L3
- statistically calculated L1
- Pulstransittime ED Ejectionduration RT Return time
- Brachialis systolic blood pressure
- Brachialis medium blood pressure
- Brachialis diastolic blood pressure
- Brachialis pulse pressure
- Brachialis augmentation pressure
- Aorta systolic blood pressure
- Aorta medium blood pressure
- Aorta diastolic blood pressure
- Aorta pulse pressure
- Aorta augmentation pressure
- Pulse Wave Velocity ba(1)=(L3---L1)/PTT
- Pulse Wave Velocity ao=(2xL2)/RT
- Pulse Wave Velocity cf (SphygmoCor®approximated)
- Aortic Aix
- Aortic AIx above normal heart rate of 75 bpm
- Brachial AIx
- Right brachial occlusion pressure
- Right brachial systolic blood pressure
- Right brachial median blood pressure
- Right brachial diastolic blood pressure
- Right brachial pulse pressure
- Right occlusion pressure ankle
- Right systolic blood pressure ankle
- Right median blood pressure ankle
- Right diastolic blood pressure ankle
- Right pulse pressure ankle
- Left brachial occlusion pressure
- Left brachial systolic blood pressure
- Left brachial median blood pressure
- Left brachial diastolic blood pressure
- Left brachial pulse pressure
- Left occlusion pressure ankle
- Left systolic blood pressure ankle
- Left median blood pressure ankle
- Left diastolic blood pressure ankle
- Left pulse pressure ankle
- Right ABI value
- Left ABI value
